# Sex-Specific Effects of Diets High in Unsaturated Fatty Acids on Spatial Learning and Memory in Guinea Pigs

**DOI:** 10.1371/journal.pone.0140485

**Published:** 2015-10-15

**Authors:** Matthias Nemeth, Eva Millesi, Karl-Heinz Wagner, Bernard Wallner

**Affiliations:** 1 Department of Behavioural Biology, Faculty of Life Sciences, University of Vienna, Vienna, Austria; 2 Department of Nutritional Sciences, Faculty of Life Sciences, University of Vienna, Vienna, Austria; 3 Department of Anthropology, Faculty of Life Sciences, University of Vienna, Vienna, Austria; Hôpital Robert Debré, FRANCE

## Abstract

Unsaturated fatty acids (UFAs), including omega-3, omega-6 polyunsaturated and omega-9 monounsaturated fatty acids, are essential components and modulators of neuromembranes and may affect various aspects of physiology and cognition. UFAs are suggested to positively affect spatial learning and memory and also to diminish the negative consequences of physiological stress on cognitive abilities. Due to pronounced sex differences in neurophysiological functions, we hypothesize that these UFA-related effects might differ between male and female individuals. We therefore determined the effects of dietary UFAs on cognitive performances in a radial-Y-maze in male and female guinea pigs in relation to saliva cortisol concentrations, a marker for physiological stress. Animals were assigned to four treatment groups and maintained on diets enriched in either chia seeds (omega-3), walnuts (omega-6), or peanuts (omega-9), or a control diet. Female learning abilities throughout a three-day learning phase were positively affected by omega-3 and omega-9, as determined by a decreasing latency to pass the test and the number of conducted errors, while males generally showed distinct learning abilities, irrespective of the diet. A sex difference in learning performances was found in the control group, with males outperforming females, which was not detected in the UFA-supplemented groups. This was paralleled by significantly increased saliva cortisol concentrations in males throughout the cognition test compared to females. Three days after this learning phase, UFA-supplemented males and all females showed unchanged performances, while control males showed an increased latency and therefore an impaired performance. These results were corroborated by pronounced differences in the plasma UFA-status, corresponding to the different dietary treatments. Our findings indicate sex-specific effects of dietary UFAs, apparently enhancing spatial learning abilities only in females and protecting males from long-term memory impairment, while male learning abilities seem to be more strongly affected by an acute physiological stress response to the maze task.

## Introduction

Unsaturated fatty acids (UFAs) are important components of neuronal cell membranes and promote several brain-mediated functions by influencing the membrane fluidity and myelinization. This may subsequently affect enzyme activities, receptor densities, or the release and reuptake of neurotransmitters and therefore brain-related physiological and cognitive functions [1,2]. The most frequent and biologically most active UFAs in the mammalian brain are the omega-3 (n-3) and omega-6 (n-6) long-chain polyunsaturated fatty acids (LC-PUFAs) eicosapentaenoic acid (EPA, 20:5 n-3), docosahexaenoic acid (DHA, 22:6 n-3), and arachidonic acid (AA, 20:4 n-6), and the omega-9 (n-9) monounsaturated fatty acid (MUFA) oleic acid (OA, 18:1 n-9) [3,4]. The LC-PUFAs are almost exclusively derived from their essential dietary precursors α-linolenic acid (ALA, 18:3 n-3) and linoleic acid (LA, 18:2 n-6) [5]. Also the majority of OA originates from the diet [6]. Although the conversion of plasma-derived ALA and LA to the respective LC-PUFAs in the brain is very low, relatively little amounts of the dietary precursors are required for a sufficient metabolic synthesis of the important LC-PUFAs [7]. Due to the lack of entire *de novo* synthesis in mammalian metabolism pathways, adequate dietary intakes are required to maintain the neuromembrane UFA-status and to prevent serious developmental dysfunctions [8].

Modifications in the dietary intake of UFAs affect the fatty acid composition in the brain, which may further influence cognitive abilities. Deficiencies in the essential n-3 precursor ALA impaired spatial learning abilities in the Barnes maze in rats and mice, which was related to decreased DHA brain concentrations [9,10]. In contrast, rats subjected to diets enriched in purified DHA or high in ALA showed improved cognitive performances in radial-arm-maze and T-maze tasks [11,12]. Epidemiological studies also revealed positive effects of n-9 MUFAs on cognitive abilities and their important role in preventing cognitive decline in the elderly [13,14]. The impact of n-6 fatty acids on cognitive functions is less studied. Nonetheless, they seem to evoke opposite effects, namely impaired learning abilities associated with higher n-6 LC-PUFA concentrations in the brain [15]. These cognitive influences are directly related to changes in neurotransmitter concentrations [10], lipid peroxidation [11], and most importantly structural and functional changes in the hippocampus, the major brain area involved in learning and memory consolidation [12].

Due to a very high density of glucocorticoid receptors, hippocampus-dependent cognitive functions can also be strongly affected by physiological stress responses and the related increase in glucocorticoid concentrations, such as that of cortisol [16,17]. Glucocorticoids are important hormonal factors released by the adrenal glands in response to a stress-related activation of the hypothalamic-pituitary-adrenal (HPA) axis. This entails physiological, metabolic, and behavioral actions that enable an organism to cope with prevalent stressors and restore body homeostasis [18]. In the long-term, elevated glucocorticoid concentrations may result in metabolic dysfunctions and impaired behavioral and cognitive performances [16]. Diets high in UFAs, however, can diminish the mostly negative effects of high stress loads. In rats subjected to prolonged stress, the administration of n-3 LC-PUFAs not only reduced glucocorticoid concentrations but also counteracted stress-related anxiety, depression, and cognitive impairments in the Morris water maze [19]. Similar effects have also been shown for a diet enriched in ALA [20]. In socially confronted guinea pigs, diets high in ALA and LA were effective in keeping cortisol concentrations at lower levels and increasing locomotive activity compared to untreated control animals [21]. These effects were the same in males and females. Nonetheless, as physiological stress responses usually differ between sexes, cognitive abilities in male and female individuals can be differently affected by glucocorticoid secretion rates [22].

A meta-analysis on cognitive sex differences in rats and mice revealed a general male advantage, but also possible species-specific effects [23]. Studies on rats indicate that such sex differences are related to acute (short-term) and chronic (long-term) stress responses [24,25]. In male rats, acute stress during the performance in a maze task was suggested to be the reason for improved cognitive abilities compared to females, while cognitive performances in females might even be facilitated by chronic stress. Such a sex difference would not be surprising because males and females differ in neurophysiological functions and in structural aspects of various brain areas [26]. Based on the known sex differences, we hypothesize that cognitive abilities and the modulatory influences of glucocorticoids are differently affected by dietary UFAs in males and females. In our study species, the domestic guinea pig (*Cavia aperea f*. *porcellus*), studies on cognitive abilities are rare and no obvious sex differences were found so far [27,28]. There is also a lack of studies on the effects of dietary UFAs and physiological stress loads on cognitive performances in this species. We therefore determined and compared the effects of diets high in n-3, n-6, and n-9 UFAs on cognitive performances in a radial-Y-maze in relation to saliva cortisol concentrations in male and female guinea pigs.

## Methods

### Ethical statement

Experiments were conducted in accordance with EU Directive 2010/63/EU for animal experiments and the Austrian laws for animal experiments and animal keeping. The study has been checked and approved by the internal board on animal ethics and experimentation of the Faculty of Life Sciences, University of Vienna (# 2014–005), and permitted by the Austrian Federal Ministry of Science and Research (BMWF-66.006/0024-II/3b/2013).

### Animals and housing conditions

Eighty domestic guinea pigs (40 males and 40 females) from a heterogeneous, multi-colored stock were used for this experiment. Their mean age was 21.6 ± 12.6 months, their weight 781.2 ± 192.1 g. All animals were therefore adult and at the same developmental level. The animals were bred at the Department of Behavioural Biology at the University of Vienna and originated from an established stock of guinea pigs from different breeders in Austria. All animals were sexually intact and accustomed to daily contact with humans. Prior to the experiments, animals were kept in socially established single-sexed groups. The floor of each enclosure was covered with standard woodchip bedding material. Each enclosure for males and females was environmentally enriched with shelters and platforms. The daily provided food consisted of guinea pig pellets (Altromin 3023, Altromin Spezialfutter GmbH & Co. KG, Lage, Germany), fresh vegetables, and hay. Guinea pig pellets and water were always available *ad libitum*. A light-dark cycle of 12/12 h with lights on at 07:00 a.m. and 22 ± 2°C were maintained throughout.

Prior to the experiment, animals were singly transferred from their social groups to cages (100 cm × 60 cm × 45 cm) in a separate test room. The floor of each cage was covered with woodchip bedding material and a shelter was provided. These social isolations should prevent behavioral or physiological influences that could be caused by social interactions. Animals were randomly allocated to four different groups (10 males and 10 females per group), each receiving a group-specific diet. Due to logistical reasons, experiments were carried out in 10 consecutive runs, with four males and four females (one male and one female per group) being tested per run. Additionally, age, bodyweight, and saliva cortisol concentrations were measured prior to the experiment and compared between experimental groups and sexes in order to exclude these variables as possible bias of the random group allocation (total model statistics: age *R* ^*2*^ = 0.069, *F*_7,71_ = 0.748, *p* = 0.633; bodyweight *R* ^*2*^ = 0.038, *F*_7,71_ = 0.401, *p* = 0.899; saliva cortisol *R* ^*2*^ = 0.046, *F*_7,71_ = 0.486, *p* = 0.842). The estrous cycle of each female was monitored by daily visual inspections of the vaginal closure prior to and during the experiment to ensure that no female was receptive during the cognition test.

### Experimental diets

Throughout the experiment the daily provided standard food consisted of 25 g guinea pig pellets, 25 g fresh vegetables (cucumber and carrot), and 5 g hay. These amounts were determined beforehand to reach the daily requirements of individually kept guinea pigs and were completely consumed by all animals in the experiment. Water was available *ad libitum*. The daily UFA-supplementation took place during the standard feeding procedures using three natural sources high in different UFAs and low in saturated fatty acids (SFAs): chia seeds (*Salvia hispanica*) are high in the n-3 ALA (17.8 g ALA, 5.8 g LA, 2.2 g OA, 3.3 g total SFAs per 100 g chia seeds), walnuts (*Juglans regia*) are high in the n-6 LA (9.1 g ALA, 38.1 g LA, 8.8 g OA, 6.1 g total SFAs per 100 g walnuts), and peanuts (*Arachis hypogaea*) are high in the n-9 OA (<0.1 g ALA, 15.6 g LA, 23.8 g OA, 8.6 g total SFAs per 100 g peanuts); information is based on the US Department of Agriculture (USDA) National Nutrient Database. For each animal 500 mg of either chia seeds (chia group), walnuts (walnut group) or peanuts (peanut group) per kg bodyweight were finely crushed, dissolved in 500 μl water, and administered orally using 1 ml syringes. The control group was supplemented with 1 ml pure water instead.

The daily provided standard food and supplementation yielded the following percentages of fatty acids (percentage on total fatty acids per dietary group): chia group: 18.4% ALA, 46.3% LA, 18.7% OA, 16.6% SFAs; walnut group: 9.8% ALA, 56.3% LA, 18.9% OA, 15% SFAs; peanut group: 4.9% ALA, 47.1% LA, 30.1% OA, 17.9% SFAs; control group: 7.1% ALA, 53.4% LA, 21.6% OA, 17.9% SFAs (calculations based on manufacturer’s information for Altromin 3023 and the USDA National Nutrient Database). Further details regarding the final nutrient compositions of each dietary group were described earlier by Nemeth et al. [21].

The applied procedure and supplemented amounts of UFA-sources with final fatty acid concentrations corresponds to previous studies in rats where the oral administration of purified DHA and walnuts has successfully been proved [11,29]. The same dosage of 500 mg/kg bodyweight has also been shown to significantly affect the fatty acid status in guinea pigs which was also related to physiological and behavioral changes [21].

### Experimental procedure

**Fig 1:**
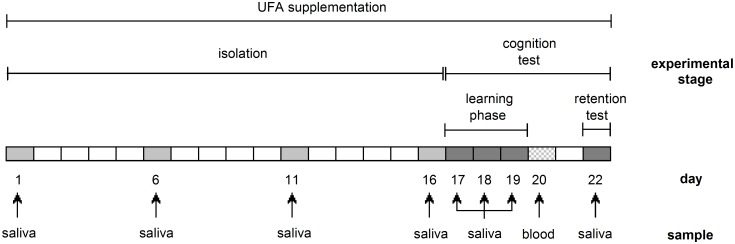
Experimental time-line for the whole experimental procedure (22 days).

The experimental procedure per animal lasted 22 days (Fig 1). This short-term UFA-supplementation procedure was chosen as dietary UFAs are relatively fast metabolized and integrated into neuromembranes [30], with distinct physiological and behavioral influences after two to three weeks of supplementations [20,21]. During the first 16 days of isolation, animals remained in their cages continuously. This was followed by a cognition test for spatial learning and memory abilities. As guinea pigs encode spatial information very quickly and the third day of learning represents some kind of cut-off point for behavioral performances [28,31], animals were subjected to the maze task for three days of learning, the learning phase (days 17–19). To determine long-term memory abilities, a single test was carried out three days after the learning phase again (day 22), the retention test. This delay of three days corresponds to the validated protocol by Machatschke et al. for studying spatial learning and memory in guinea pigs [31]. Tests were always conducted between 09:00 a.m. and 11:00 a.m. Feeding procedures were carried out daily at 11:00 a.m. All animals were returned to their single-sexed groups after day 22.

Spatial learning and memory abilities were tested in a radial-Y-maze designed by Millesi et al. [32] and adopted for guinea pigs by Machatschke et al. [31]. The maze was built of wooden panels (40 cm in height) and consisted of a central area (70 cm × 70 cm) which was connected to four regular arms (60 cm in length, 20 cm in width) that originated from the corners of the central area, each splitting in two separate arms (each 50 cm in length, 20 cm in width) like a Y. Different furniture, including shelves and tables with laboratory and electrical equipment, and doors surrounded the maze, serving as visuospatial extra-maze cues for orientation. For each animal, one of the eight end-arms was baited randomly with a single piece of cucumber as reward. The baited arm remained the same for each single animal throughout the cognition test, including the learning phase (days 17–19) and the retention test (day 22). On each day of the cognition test, animals had to perform once per day in the maze. The maximum time of daily performance per individual was 10 minutes, whether the reward was found or not. Prior to each animal being tested, the floor and walls of the maze were cleaned with a diluted vinegar-based cleaner, containing 5% acetic acid, to remove olfactory influences. Each single performance was recorded with a video camera located at the ceiling.

To determine cortisol concentrations, a marker for physiological stress reactions, saliva samples were collected four times during the 16-day isolation period (days 1, 6, 11, 16) and on each day of the maze task, directly after the performance of each animal (days 17, 18, 19, 22). Saliva was collected by inserting a cotton bud into the animals’ cheek pouch for 1 minute. This method was developed as a non-invasive option to determine the HPA-axis activity in guinea pigs [33]. After centrifugation (14000 rpm, 17968 × g, 10 minutes) samples were stored at -20°C until further analysis.

Blood samples were collected after the learning phase (day 20) to determine plasma fatty acid patterns as a proof for the successful uptake from the diet. Marginal ear veins were punctured and approximately 300 μl of the flowing blood was collected with heparinized (5000 units) micropipettes [34]. Plasma was separated by centrifugation (14000 rpm, 17968 × g, 10 minutes) and stored at -20°C until further analysis.

### Behavioral analysis

Using the Observer XT 10 software (Version 10.5.572, Noldus, Wageningen, the Netherlands), three different behavioral parameters were measured: (1) Latency: the period of time from release into the maze until the reward was reached. If an animal failed to find the reward, 10 minutes were recorded. (2) Error-rate: the number of wrong arm-entries until the reward was found or, if it was not found, until the maximum time of 10 minutes elapsed. (3) Percentage of movement: the percentage of time an animal spent moving around until the reward was found or, if it was not found, until the maximum time of 10 minutes elapsed.

### Saliva cortisol analysis

After thawing, 20 μl of the saliva samples were diluted 1:40 and cortisol concentrations measured in 10 μl duplicates using biotin-strepdavidin enzyme-linked immunoassay [35]. A cortisol-specific antibody was used (University of Veterinary Medicine, Vienna, Austria), which cross-reacted with the following steroids: 4-pregnene-11β,21-diol-3,20-dione 6.2%; 4-pregnene-11β,17α,21-triol-3,20,dione 100%; 5α-pregnane-11β,17α,21-triol-3,20,dione 4.6%; 5α-pregnane-3α,11β,17α,21-tetrol-20-one 0.8%; 5β-pregnane 3α,11β,17α,21-tetrol-20-one 0.1%; all other steroids cross-reacted < 0.01%. Intra- and interassay coefficients of variance were 8.1% and 13.4%, respectively.

### Plasma fatty acid analysis

Plasma fatty acids were analyzed using gas chromatography, following the protocol of Wagner et al. [36]. 1 ml methanolic NaOH, containing butylated hydroxytoluene to prevent oxidation, was added to 100 μl plasma for fatty acid transesterification. Afterwards, 1 ml 14% boron-trifluoride-methanol was added to obtain fatty acid methyl esters (FAMES). FAMES were extracted into 500 μl hexane four times, vaporized, and re-dissolved in hexane. Thereafter, FAMES were separated by an Rtx-2330 30 m × 0.25 mm i.d. silica column using an Auto-System gas chromatograph with flame ionization detector (Perkin Elmer, USA). 1 μl of prepared samples was injected at a temperature of 250°C and detected at 270°C; helium was used as carrier gas. Fatty acids were identified by a 37 component FAME Mix Standard (Supelco, Bellafonte, USA). Peak integration was carried out using the TotalChrom Workstation 6.3.0 software (PE Nelson, Perkin Elmer, USA).

### Statistical analysis

Statistics were performed using R 3.0.0 [37] and the additional packages ‘nlme’ [38] for calculating linear mixed effect models (LMEs) and ‘phia’ [39] for post-hoc interaction analyses. LMEs were used in the case of repeated measurements (cognitive parameters and saliva cortisol concentrations) and favored over repeated measures ANOVAs, which constitutes an alternative way of analyzing the current data. This is because LMEs enable a broader range of model fitting (stepwise deletion of non-significant interactions and main effects).

The three cognitive parameters (latency, errors, movement) during the learning phase were analyzed by applying LMEs with ‘group’ (chia, walnut, peanut, control), ‘sex’ (males, females), and ‘day’ (day1, day2, day3) as fixed factors and single individuals as random factors to control for the repeated measurements. The retention test was analyzed in relation to the last day of the learning phase by calculating LMEs with ‘group’ (chia, walnut, peanut, control), ‘sex’ (males, females), and ‘day’ (day3, retention test) as fixed factors and again single individuals as random factors. The starting models included all possible interactions between the fixed factors (full models). Post-hoc interaction analyses were carried out on the full models to analyze the daily changes in the cognitive parameters of single groups within both sexes, without group or sex comparisons. Models were then fitted based on the Akaike information criterion (AIC). Post-hoc interaction analyses with bonferroni corrections were carried out for further analyses of the fitted models. In case of significant three-way interactions, the daily changes in the cognitive parameters and performances on single days were compared between groups within each sex and between sexes within each group.

For analysis of saliva cortisol concentrations, an LME was calculated including ‘group’ (chia, walnut, peanut, control), ‘sex’ (males, females), ‘day’ (days 1, 6, 11, 16, 17, 18, 19, 22), and ‘experimental stage’ (isolation, cognition test) as fixed factors and single individuals as random factor. Plasma fatty acids were analyzed by applying two-way analysis of variance (ANOVAs), including the predictors ‘group’ (chia, walnut, peanut, control) and ‘sex’ (males, females). After model fitting (AIC), post-hoc interaction analyses with bonferroni corrections were carried out on significant effects.

Model assumptions were checked using the Shapiro-Wilk normality test and Levene’s Test for homogeneity of variance and by visual inspection of residual and fitted value plots. Some of the response variables had to be transformed by applying the natural logarithm or the second or third root, but were back-transformed for visualization of significant effects. In the case of significant high-term interaction effects including the predictors ‘group’ and ‘sex’ (e.g. ‘Group:Sex:Day’), for simplification purposes the results are visualized separately for the sexes. Only the highest significant terms or interactions of the calculated models which remained in the fitted models are considered in the result section; for full model statistics see S1 Table. The level of significance was set at *p* ≤ 0.05.

## Results

### Learning phase

**Fig 2:**
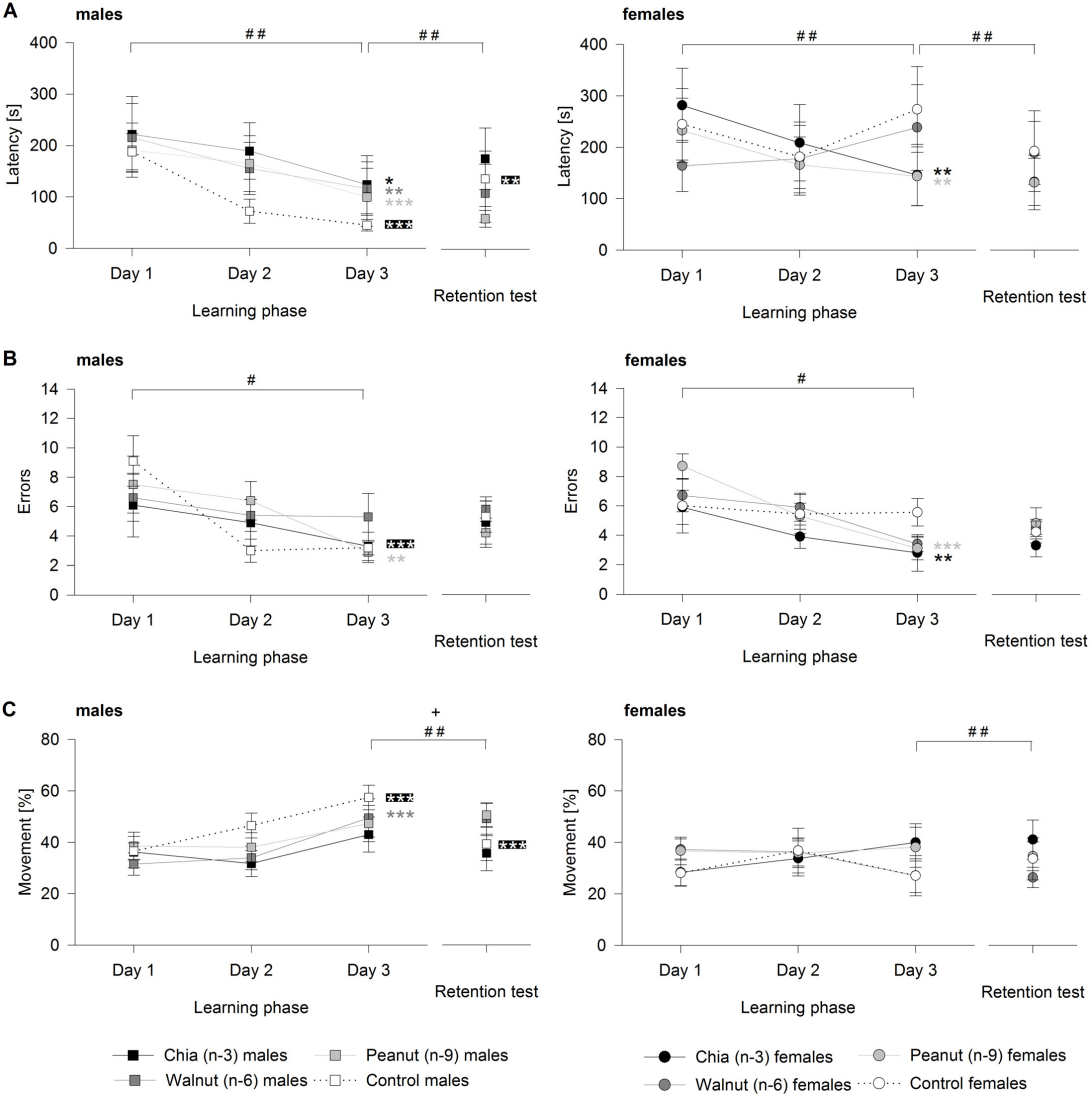
Behavior of male and female groups during the spatial cognition test. (A) Latency time to pass the test for males and females. (B) Number of conducted errors for males and females until the test was passed. (C) Percentage of movement for males and females until the test was passed. Values are means ± SEM. Asterisks next to the symbols on day 3 of the learning phase indicate significant changes in the time course throughout the learning phase for single male and female groups; asterisks next to the symbols of the retention test indicate significant changes compared to day 3 of the learning phase for single male and female groups: * *p* ≤ 0.05, ** *p* ≤ 0.01, *** *p* ≤ 0.001 (chia, walnut, peanut, control). Significant differences in the time course between single male and/or female groups throughout the learning phase, as well as from day 3 of the learning phase to the retention test are indicated as following: # *p* ≤ 0.05, ## *p* ≤ 0.01 comparing control males and control females; + *p* ≤ 0.05 comparing control males and peanut males.

Regarding the latency a significant three-way interaction of group, sex, and day was found during the learning phase (*F*_6,142_ = 2.121, *p* = 0.050). All male groups showed a significant decrease from day to day throughout the learning phase (chia males: *χ* ^*2*^ = 6.963, *p* = 0.031; walnut males: *χ* ^*2*^ = 11.258, *p* = 0.004; peanut males: *χ* ^*2*^ = 13.784, *p* = 0.001; control males: *χ* ^*2*^ = 28.391, *p* < 0.001). In contrast, only females of the chia and peanut groups showed similar patterns (chia females: *χ* ^*2*^ = 11.210, *p* = 0.004; walnut females: *χ* ^*2*^ = 0.621, *p* = 0.733; peanut females: *χ* ^*2*^ = 9.984, *p* = 0.007; control females: *χ* ^*2*^ = 1.551, *p* = 0.461) (Fig 2A). The daily changes in the latency did not differ significantly between groups within both sexes (males: *χ* ^*2*^ = 10.378, *p* = 0.219; females: *χ* ^*2*^ = 10.436, *p* = 0.215). Within the groups, a significant sex difference occurred only in the control group, caused by the decrease in males and no change in females, while no differences were found in the remaining groups (chia group: *χ* ^*2*^ = 2.111, *p* = 1; walnut group: *χ* ^*2*^ = 4.961, *p* = 0.335; peanut group: *χ* ^*2*^ = 0.714, *p* = 1; control group: *χ* ^*2*^ = 13.153, *p* = 0.006). The latency on day three of the learning phase was significantly lower in control males versus control females (*χ* ^*2*^ = 12.903, *p* = 0.004; *p* ≥ 0.581 for all further pairwise comparisons), while no differences were found between groups and/or sexes on the remaining days (day 1: *χ* ^*2*^ = 1.309, *p* = 1: day 2: *χ* ^*2*^ = 3.651, *p* = 0.905).

Similar to the latency, a significant three-way interaction of group, sex, and day was found for the number of conducted errors (*F*_6,142_ = 2.374, *p* = 0.032). Males of the peanut and control groups showed a decreasing number of errors from day to day, while no changes occurred in the other male groups (chia males: *χ* ^*2*^ = 1.694, *p* = 0.429; walnut males: *χ* ^*2*^ = 1.248, *p* = 0.536; peanut males: *χ* ^*2*^ = 10.412, *p* = 0.005; control males: *χ* ^*2*^ = 18.530, *p* < 0.001). In females, similar declines were found for the chia and peanut groups (chia females: *χ* ^*2*^ = 9.224, *p* = 0.010; walnut females: *χ* ^*2*^ = 4.601, *p* = 0.100; peanut females: *χ* ^*2*^ = 18.099, *p* < 0.001; control females: *χ* ^*2*^ = 0.140, *p* = 0.932) (Fig 2B). Within the sexes, no significant differences between groups occurred from day to day (males: *χ* ^*2*^ = 13.140, *p* = 0.081; females: *χ* ^*2*^ = 11.368, *p* = 0.155). Nonetheless, the daily decline in the number of errors differed significantly in control males versus control females, while no such sex differences occurred in the other groups (chia group: *χ* ^*2*^ = 1.894, *p* = 1; walnut group: *χ* ^*2*^ = 0.621, *p* = 1; peanut group: *χ* ^*2*^ = 2.130, *p* = 1; control group: *χ* ^*2*^ = 9.732, *p* = 0.031). The number of conducted errors did not differ between groups and/or sexes on any of the three days of the learning phase (day 1: *χ* ^*2*^ = 5.743, *p* = 0.374; day 2: *χ* ^*2*^ = 1.988, *p* = 1; day 3: *χ* ^*2*^ = 1.980, *p* = 1).

The third parameter, the percentage of movement, revealed a significant difference from day to day only regarding sexes (*F*_2,142_ = 4.979, *p* = 0.008), but no significant group-related effects. Males generally showed increasing movement, whereas no such effect was detected in females (males: *χ* ^*2*^ = 30.129, *p* < 0.001; females: *χ* ^*2*^ = 1.480, *p* = 0.954). Considering single male and female groups revealed that only males of the control and walnut groups showed increased movement during the learning phase, with no further significant changes (chia males: *χ* ^*2*^ = 4.443, *p* = 0.108; walnut males: *χ* ^*2*^ = 13.165, *p* = 0.001; peanut males: *χ* ^*2*^ = 3.744, *p* = 0.154; control males: *χ* ^*2*^ = 15.109, *p* < 0.001; chia females: *χ* ^*2*^ = 4.676, *p* = 0.097; walnut females: *χ* ^*2*^ = 4.381, *p* = 0.111; peanut females: *χ* ^*2*^ = 0.183, *p* = 0.912; control females: *χ* ^*2*^ = 3.579, *p* = 0.167) (Fig 2C).

### Retention test

Comparing the latency on the last day of the learning phase and in the retention test revealed a significant three-way interaction of group, sex, and day (*F*_3,71_ = 3.125, *p* = 0.031). A significant increase in the latency from the last day of the learning phase to the retention test was found for control males, while the latency did not change in all other male and female groups (chia males: *χ* ^*2*^ = 1.751, *p* = 0.186; walnut males: *χ* ^*2*^ = 0.006, *p* = 0.937; peanut males: *χ* ^*2*^ = 0.084, *p* = 0.772; control males: *χ* ^*2*^ = 8.773, *p* = 0.003; chia females: *χ* ^*2*^ = 0.014, *p* = 0.905; walnut females: *χ* ^*2*^ = 0.187, *p* = 0.666; peanut females: *χ* ^*2*^ = 0.843, *p* = 0.359; control females: *χ* ^*2*^ = 2.042, *p* = 0.153) (Fig 2A). This increase in the latency in control males, however, did not differ from the other male groups, nor was such a difference detected between females (males: *χ* ^*2*^ = 6.781, *p* = 0.158; females: *χ* ^*2*^ = 3.082, *p* = 0.758). Within the groups, the change in latency differed only between control males and females, reflecting the strong increase in males (chia group: *χ* ^*2*^ = 1.040, *p* = 1; walnut group: *χ* ^*2*^ = 0.131, *p* = 1; peanut group: *χ* ^*2*^ = 0.730, *p* = 1; control group: *χ* ^*2*^ = 9.457, *p* = 0.008). Therefore, the latency did not differ between any groups and/or sexes in the retention test (*χ* ^*2*^ = 3.734, *p* = 0.583).

The number of conducted errors generally increased comparing the last day of the learning phase and the retention test (*F*_1,78_ = 7.744, *p* = 0.007). This did not differ between groups and/or sexes, resulting in an elimination of each group- and sex-related effect during model simplification. Considering the error-rate of single male and female groups in the retention test, no differences were detected compared to the learning phase (chia males: *χ* ^*2*^ = 0.983, *p* = 0.322; walnut males: *χ* ^*2*^ = 1.862, *p* = 0.172; peanut males: *χ* ^*2*^ = 1.061, *p* = 0.303; control males: *χ* ^*2*^ = 3.050, *p* = 0.081; chia females: *χ* ^*2*^ = 0.988, *p* = 0.320; walnut females: *χ* ^*2*^ = 0.927, *p* = 0.336; peanut females: *χ* ^*2*^ = 3.315, *p* = 0.069; control females: *χ* ^*2*^ = 0.812, *p* = 0.368) (Fig 2B).

Regarding the percentage of movement, a significant three-way interaction of group, sex, and day was detected (*F*_3,71_ = 3.395, *p* = 0.023). A decrease from the last day of the learning phase to the retention test occurred only in control males (chia males: *χ* ^*2*^ = 2.047, *p* = 0.153; walnut males: *χ* ^*2*^ = 0.006, *p* = 0.938; peanut males: *χ* ^*2*^ = 0.384, *p* = 0.536; control males: *χ* ^*2*^ = 12.455, *p* < 0.001; chia females: *χ* ^*2*^ = 0.050, *p* = 0.823; walnut females: *χ* ^*2*^ = 0.018, *p* = 0.895; peanut females: *χ* ^*2*^ = 0.445, *p* = 0.505; control females: *χ* ^*2*^ = 1.499, *p* = 0.221) (Fig 2C). No differences comparing the learning phase and the retention test were detected between female groups, but it differed significantly between males (males: *χ* ^*2*^ = 10.012, *p* = 0.037; females: *χ* ^*2*^ = 1.923, *p* = 1). The decrease in control males differed significantly to peanut males (*χ* ^*2*^ = 8.606, *p* = 0.040; *p* ≥ 0.176 for all further pairwise comparisons) and did also differ significantly from control females, whereas no sex differences occurred in the remaining groups (chia group: *χ* ^*2*^ = 1.368, *p* = 0.969; walnut group: *χ* ^*2*^ = 0.002, *p* = 1; peanut group: *χ* ^*2*^ = 0.827, *p* = 1; control group: *χ* ^*2*^ = 11.004, *p* = 0.004). Finally, the percentage of movement did not differ between any groups and/or sexes in the retention test (*χ* ^*2*^ = 5.791, *p* = 0.245).

### Saliva cortisol concentrations

Significant differences in saliva cortisol concentrations were detected between groups and sexes during the isolation period and the cognition test (Group:Stage *F*_3,542_ = 3.033, *p* = 0.029; Sex:Stage *F*_1,542_ = 29.288, *p* < 0.001). Within both experimental stages (isolation and cognition test), cortisol levels differed only marginally between the single days, with no differences between single groups or sexes (see S1 Table).

**Fig 3:**
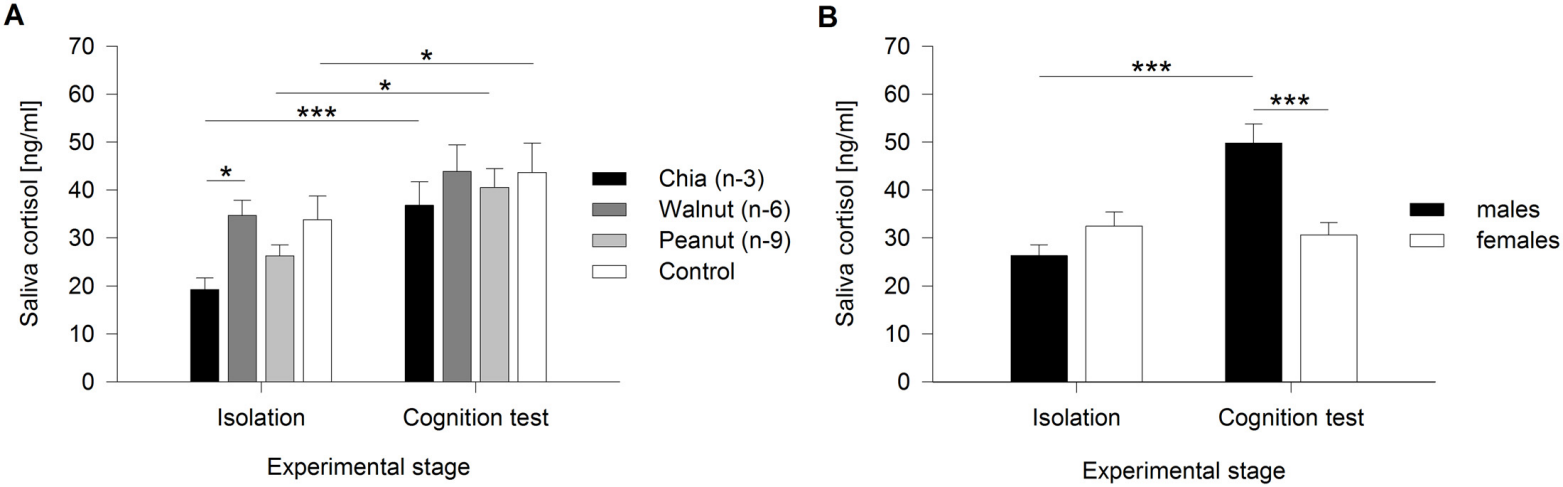
Saliva cortisol concentrations throughout the isolation period and the cognition test. (A) Saliva cortisol [ng/ml] for single groups (both sexes combined) based on the significant interaction of ‘group’ and ‘stage’. (B) Saliva cortisol [ng/ml] for the sexes (all four groups combined) based on the significant interaction of ‘sex’ and ‘stage’. Values are means ± SEM. * *p* ≤ 0.05, ** *p* ≤ 0.01, *** *p* ≤ 0.001.

A significant difference between groups occurred during isolation, whereas no such difference was detected throughout the cognition test (isolation: *χ* ^*2*^ = 11.209, *p* = 0.021; cognition test: *χ* ^*2*^ = 1.065, *p* = 1). During isolation, cortisol levels of the walnut group exceeded those of the chia group (*χ* ^*2*^ = 10.582, *p* = 0.014; *p* ≥ 0.200 for all further pairwise comparisons). Saliva cortisol concentrations, however, were higher throughout the cognition test in the chia, peanut, and control groups compared to isolation (chia group: *χ* ^*2*^ = 25.253, *p* < 0.001; walnut group: *χ* ^*2*^ = 0.589, *p* = 1; peanut group: *χ* ^*2*^ = 9.023, *p* = 0.011; control group: *χ* ^*2*^ = 7.145, *p* = 0.030) (Fig 3A).

Cortisol concentrations did not differ between males and females during isolation (*χ* ^*2*^ = 1.611, *p* = 0.409), but were higher in males during the cognition test (*χ* ^*2*^ = 12.950, *p* < 0.001). Correspondingly, cortisol levels in males were elevated during the cognition test compared to isolation, while female levels did not change (males: *χ* ^*2*^ = 62.918, *p* < 0.001; females: *χ* ^*2*^ = 0.051, *p* = 1) (Fig 3B).

### Plasma fatty acids

Plasma fatty acids were affected only by group and/or sex, but no significant interactions of group and sex were detectable. Table 1 therefore only shows the most prominent plasma fatty acids for each experimental group (for a full list of plasma fatty acids for males and females of each groups see S2 Table). Irrespective of group and sex, LA (18:2 n-6) was the most abundant plasma fatty acid, followed by OA (18:1 n-9), the SFAs palmitic acid (16:0) and stearic acid (18:0), and ALA (18:3 n-3). Those five fatty acids accounted for 90.34 ± 0.32% of total detected fatty acids in plasma, with no differences between groups or sexes (group: *F*_3,67_ = 1.373, *p* = 0.259; sex: *F*_1,67_ = 0.230, *p* = 0.634; group:sex: *F*_3,67_ = 0.653, *p* = 0.584). Nonetheless, the percentages of single fatty acids differed considerably between the groups. The chia group showed the highest percentage of ALA, the peanut group the lowest value. The same pattern was found for total n-3 fatty acids. The walnut and peanut groups exhibited the highest percentage of LA and OA, respectively. Therefore, the walnut group was highest in total PUFAs with total n-6 dominating, while the peanut group was highest in MUFAs with n-9 dominating. Only minor differences were detected in single SFAs, whereas total SFAs were highest in the control group, which differed significantly from the walnut group. Therefore, the chia, walnut, and peanut groups also showed the highest plasma n-6:n-3, P:S, and M:S ratios, respectively (Table 1).

**Table 1:** Most prominent plasma fatty acids (% of total fatty acids) for the experimental groups.

Fatty Acids	Group			
	Chia (n-3)	Walnut (n-6)	Peanut (n-9)	Control
16:0	14.62 ± 0.33	12.99 ± 0.83	13.52 ± 0.40	14.82 ± 0.74
18:0	11.70 ± 0.32	10.92 ± 0.51	11.39 ± 0.29	12.24 ± 0.68
18:1 n-9	11.97 ± 0.72 ^a^	12.69 ± 0.59 ^a^	17.16 ± 0.68 ^b^	12.48 ± 0.29 ^a^
18:2 n-6	43.37 ± 1.09 ^a^	48.05 ± 1.14 ^b^	44.22 ± 0.91 ^a^	44.40 ± 0.98 ^a^
18:3 n-3	9.58 ± 0.60 ^a^	6.20 ± 0.30 ^b^	4.07 ± 0.19 ^c^	5.23 ± 0.26 ^b^
**Totals**				
n-9	12.12 ± 0.72 ^a^	12.93 ± 0.58 ^a^	17.52 ± 0.71 ^b^	12.75 ± 0.29 ^a^
n-6	46.36 ± 1.07 ^a^	50.92 ± 1.11 ^b^	47.58 ± 0.89 ^a^	47.75 ± 0.99 ^a^
n-3	10.26 ± 0.56 ^a^	6.79 ± 0.31 ^b^	4.78 ± 0.24 ^c^	6.02 ± 0.24 ^b^
PUFA	56.62 ± 0.94 ^ac^	57.71 ± 1.10 ^a^	52.36 ± 0.86 ^b^	53.77 ± 0.95 ^bc^
MUFA	13.68 ± 0.67 ^a^	15.02 ± 0.60 ^a^	19.45 ± 0.68 ^b^	14.95 ± 0.27 ^a^
UFA	70.30 ± 0.77 ^ab^	72.73 ± 1.12 ^a^	71.81 ± 0.87 ^ab^	68.71 ± 0.98 ^b^
SFA	29.70 ± 0.77 ^ab^	27.27 ± 1.12 ^a^	28.19 ± 0.87 ^ab^	31.29 ± 0.98 ^b^
**Ratios**				
n6:n3 ratio	4.82 ± 0.34 ^a^	7.87 ± 0.58 ^b^	10.46 ± 0.62 ^c^	8.21 ± 0.43 ^b^
M:S ratio	0.47 ± 0.02 ^a^	0.57 ± 0.04 ^a^	0.71 ± 0.04 ^b^	0.49 ± 0.02 ^a^
P:S ratio	1.94 ± 0.08 ^ab^	2.20 ± 0.13 ^a^	1.90 ± 0.08 ^ab^	1.77 ± 0.08 ^b^
U:S ratio	2.41 ± 0.09 ^ab^	2.77 ± 0.16 ^a^	2.60 ± 0.10 ^ab^	2.25 ± 0.10 ^b^

Different superscripts indicate significant differences between groups (p ≤ 0.05).

## Discussion

The present study investigated the modulatory effects of dietary n-3, n-6, and n-9 UFAs on spatial cognitive abilities in relation to saliva cortisol secretion rates in male and female guinea pigs. Our findings indicate a sex-specific effect of dietary UFAs: they improved learning abilities in a radial-Y-maze task only in females and protected males from long-term memory impairments. These sex-specific effects of dietary UFAs, and the general sex difference in spatial cognition observed in control animals, were significantly related to physiological stress reactions and corresponding cortisol concentrations.

Manipulating the UFA-status by supplementing chia seeds, walnuts, or peanuts as natural UFA-sources for 22 days resulted in significantly increased percentages of the corresponding fatty acids in plasma. This was ALA and total n-3 in the chia group, LA and total n-6 in the walnut group, and OA and total n-9 in the peanut group. Plasma-derived UFAs seem to be rapidly metabolized in mammalian metabolism pathways and integrated into neuromembrane phospholipids [40]. As the synthesis of LC-PUFAs and their integration into phospholipids takes place mainly in the liver [41], the plasma LC-PUFA-status remains mostly unaffected by treatments with the dietary precursors. Accordingly, this status also did not differ markedly between groups in our study. Although only small amounts of ALA and LA are converted to LC-PUFAs, they significantly contribute to the maintenance of the LC-PUFA-status in the brain [7]. Therefore, the plasma UFA-status in our animals reflects the neuromembrane UFA-status and the overall potential for UFA-related metabolic actions.

The positive effects of dietary UFAs and the plasma UFA-status on learning abilities in this study occurred mainly in females. Our results revealed a decrease in the latency to pass the test and fewer conducted errors during the three-day learning phase in females supplemented with chia seeds (n-3) and peanuts (n-9). No such effect was detected in control and walnut (n-6) females. This fully corroborates to previous findings showing positive effects of n-3 and n-9 fatty acids on cognitive functions [12,13]. The reason why exactly these types of fatty acids elicit enhanced cognitive performances remains unknown. One suggestion is that the structural and biochemical properties of UFAs differently influence the membrane fluidity and related activities of ion channels, receptors, neurotransmitters, or membrane-bound proteins, and therefore also cognitive functions [42]. Especially the ratio of n-6 and n-3 fatty acids seems to be a crucial factor regarding these effects. In this context, a dietary n-6:n-3 (ALA:LA) ratio of 4:1 has proved to best enhance learning abilities of rats in the Morris water maze [43]. The current study nearly achieved this proposed ratio of n-6:n-3 fatty acids in the plasma of the chia group. Therefore, it is not surprising that the supplementation of chia seeds as a natural ALA source improved spatial learning abilities. For n-9 fatty acids, no such effects have been shown so far, but similar effects on neurophysiological functions can be assumed as for n-3 fatty acids [44]. Our findings demonstrate that n-3 and n-9 fatty acids can both positively affect cognitive abilities in the same way. Although neurobiological influences were not investigated here, similar influences of n-3 and n-9 on neuromembrane structure and function may be indicated by this result. Nonetheless, as cognitive performances in the retention test remained unaffected in all groups, dietary UFAs apparently had no effects on long-term memory abilities in females. This result also shows that guinea pigs are generally able to encode spatial information in a radial-Y-maze even after a three-day intermission, confirming a long-lasting preservation of memory abilities in this species [31].

Our results further suggest that UFA-related effects on cognition differ between the sexes. All male groups showed a decreasing latency throughout the learning phase, indicating a learning effect in all groups. Control and peanut (n-9) males further showed a decreasing number of conducted errors, whereby control and walnut (n-6) males also showed increasing movement. Learning performances in males were therefore most pronounced in the untreated control group. This contrasts to the observed positive effects of n-3 and n-9 fatty acids on learning abilities in females and to the general knowledge of the positive effects of dietary UFAs [45]. Nonetheless, positive effects of dietary UFAs in males were observed in the retention test. Long-term memory abilities of control males were impaired in the retention test versus the learning phase, while all UFA-supplemented males showed no such differences and therefore preserved long-term memory abilities. These different effects in control and UFA-supplemented males might reflect the general fatty acid status. While the chia, walnut, and peanut groups showed highest plasma levels in total PUFAs and MUFAs, the control group revealed highest levels of SFAs. Opposite to the positive effects of dietary UFAs, a high intake of SFAs can strongly impair cognitive functions [46]. In our study the same might be true for males of the control group and the impaired performance in the retention test. Finally, different effects in males and females could also be explained by different physiological stress reactions. Especially learning abilities in males could have been more strongly affected by cortisol secretion rates than by dietary UFAs.

Only male individuals showed significantly increased saliva cortisol concentrations during the daily performance in the radial-Y-maze. Additionally, the improved learning performance of control males differed significantly from control females, which showed no learning effect at all. As previously suggested, acute stress probably caused by the task itself can have sex-specific effects on cognitive abilities [25]. Especially memory for emotionally arousing information seems to be enhanced due to increased cortisol concentrations [47], involving the hippocampus and amygdala brain areas [48]. Transferred to our findings, male guinea pigs could have been more aroused by the cognitive task than females, as reflected in increased saliva cortisol concentrations and improved learning abilities. Similar effects were shown previously in that guinea pigs that performed better in a radial-Y-maze task also had increased cortisol concentrations [31]. This would also include that the positive effects of short-term increased cortisol concentrations on male learning abilities exceeded possible influences of dietary UFAs in our study. Additionally, long-term memory abilities in the retention test could have been negatively affected by this short-term physiological stress response. In contrast, females would have remained unaffected by the task itself, but were positively affected by dietary supplementations with n-3 and n-9 fatty acids. As no differences in learning abilities between males and females occurred in the UFA-supplemented groups, we also conclude that dietary n-3, n-6, and n-9 UFAs extinguished the general sex difference found in the control group.

Short-term stress responses in the cognition test differed significantly between the sexes, but not long-term cortisol levels as measured during the isolation period. This contrasts with previous findings on general sex differences in baseline cortisol levels and the HPA-axis reactivity [24]. We also found no effects of the social isolation itself: no differences in saliva cortisol were detected between the single days during the isolation period in either sex. This underlines that social isolation is not necessarily a source of stress in a basically social-living animal, as shown previously in guinea pigs [49]. Nonetheless, baseline saliva cortisol concentrations may have been affected by dietary UFAs, as indicated by lower levels in the chia (n-3) versus walnut (n-6) group. In guinea pigs, n-3 and n-6 PUFAs can diminish cortisol secretion rates and behavioral responses due to social stress [21]. In the current study the difference in saliva cortisol concentrations during isolation was not obviously related to such behavioral differences during the cognition test. Note, however, that n-3 LC-PUFAs can for example diminish long-term stress loads and therefore stress-related behavioral and cognitive impairments [19]. Accordingly, the slightly decreased baseline cortisol levels we found in the chia group may have additionally improved the learning performance in chia females.

This study supports the findings on positive influences of dietary UFAs, especially n-3 and n-9 fatty acids, on cognitive abilities. Importantly, our results also indicate that these effects differ for males and females and that sex differences in cognitive abilities are generally related to other physiological factors, such as glucocorticoid secretion rates. As neurophysiological and cognitive functions differ in male and female individuals [50], it is not surprising that dietary UFAs and physiological stress responses can have sex-specific effects. We therefore suggest that sex differences, especially in neurobiological and physiological functions, should definitely be more strongly considered when studying the effects of dietary UFAs. This would no doubt sharpen our understanding of how dietary UFAs can improve cognitive functions and even counteract stress-related cognitive impairments.

## Supporting Information

S1 File Dataset. (PDF)

S1 Table Model statistics for behaviors of the spatial cognition test and saliva cortisol concentrations. (PDF)

S2 Table Plasma fatty acids (% on total fatty acids) for single male and female groups. (PDF)
