# Supplementary material for: Sex-Specific Effects of Diets High in Unsaturated Fatty Acids on Spatial Learning and Memory in Guinea Pigs
Source: PLoS One. 2015 Oct 15;10(10):e0140485. doi: 10.1371/journal.pone.0140485 (PMC4607430; doi:10.1371/journal.pone.0140485)
Supplement: S1 File — (PDF) [file pone.0140485.s001.pdf]

| Code | Group | Sex | Cort_Day01 | Cort_Day06 | Cort_Day11 | Cort_Day16 | Cort_Day17 |
|---|---|---|---|---|---|---|---|
| Sm1 | Chia | male | 14.81 | 36.38 | 11.43 | 12.37 | 67.65 |
| Sm2 | Chia | male | 19.07 | 7.23 | 10.58 | 20.45 | 56.62 |
| Sm3 | Chia | male | 98.15 | 30.09 | 8.72 | 17.21 | 96.97 |
| Sm4 | Chia | male | 68.61 | 29.89 | 6.26 | 9.89 | 30.89 |
| Sm5 | Chia | male | 9.58 | 2.46 | 3.45 | 3.8 | 26.93 |
| Sm6 | Chia | male | 18.98 | 12.32 | 6.62 | 10.6 | 7.72 |
| Sm7 | Chia | male | 15.75 | 10.82 | 5.88 | 14.65 | 59.37 |
| Sm8 | Chia | male | 9.06 | 18.11 | 8.03 | 9.15 | 59.15 |
| Sm9 | Chia | male | 12.2 | 1.55 | 3.11 | 0.7 | 14.4 |
| Sm10 | Chia | male | 3.95 | 2.13 | 3.18 | 41.74 | 3.04 |
| Sf1 | Chia | female | 38.26 | 108.05 | 9.45 | 50.28 | 40.29 |
| Sf2 | Chia | female | 2.57 | 13.08 | 36.39 | 98.41 | 8.28 |
| Sf3 | Chia | female | 13.83 | 53.13 | 7.86 | 11.98 | 14.38 |
| Sf4 | Chia | female | 14.79 | 58.37 | 41.71 | 21.52 | 55.81 |
| Sf5 | Chia | female | 33.82 | 13.33 | 24.83 | 26.21 | 21.94 |
| Sf6 | Chia | female | 27.2 | 14 | 2.9 | 14.24 | 6 |
| Sf7 | Chia | female | 2.94 | 4.55 | 5.21 | 5.87 | 4.24 |
| Sf8 | Chia | female | 2.77 | 55.83 | 7.73 | 5.38 | 37.23 |
| Sf9 | Chia | female | 9.93 | 3.38 | 6.55 | 6.54 | 5.47 |
| Sf10 | Chia | female | 33.89 | 6.48 | 2.53 | 12.96 | 12.34 |
| Wm1 | Walnut | male | 14.81 | 33.7 | 39.19 | 42.64 | 89.66 |
| Wm2 | Walnut | male | 9.57 | 56.43 | 31.31 | 73.45 | 46.19 |
| Wm3 | Walnut | male | 25.89 | 77.56 | 23.23 | 76.67 | 35.53 |
| Wm4 | Walnut | male | 3.98 | 48.78 | 6.59 | 14.27 | 160.41 |
| Wm5 | Walnut | male | 74.77 | 12.77 | 33.21 | 7.6 | 47.13 |
| Wm6 | Walnut | male | 5.82 | 15.06 | 12.12 | 9.18 | 1.66 |
| Wm7 | Walnut | male | 12.78 | 6.81 | 4.93 | 102.54 | 96.42 |
| Wm8 | Walnut | male | 23.36 | 98.3 | 107.75 | 95.77 | 110.81 |
| Wm9 | Walnut | male | 39.55 | 7.21 | 2.7 | 11.22 | 41.96 |
| Wm10 | Walnut | male | 42.11 | 20.26 | 39.52 | 58.77 | 8.49 |
| Wf1 | Walnut | female | 33.23 | 98.66 | 43.4 | 36.4 | 29.72 |
| Wf2 | Walnut | female | 3.17 | 4.36 | 11.03 | 40.46 | 2.77 |
| Wf3 | Walnut | female | 27.57 | 1.36 | 12.63 | 21.03 | 21.69 |
| Wf4 | Walnut | female | 25.29 | 109.22 | 44.79 | 23.03 | 16.35 |
| Wf5 | Walnut | female | 26.18 | 39.79 | 40.52 | 59.07 | 25.91 |
| Wf6 | Walnut | female | 24.27 | 75.74 | 14.24 | 42.07 | 10.2 |
| Wf7 | Walnut | female | 24.26 | 7.65 | 76.41 | 10.72 | 87.55 |
| Wf8 | Walnut | female | 10.12 | 18.87 | 11.76 | 9.5 | 13.76 |
| Wf9 | Walnut | female | 82.84 | 75.28 | 5.6 | 25.31 | 10.34 |
| Wf10 | Walnut | female | 23.92 | 33.3 | 46.73 | 28.28 | 3.95 |
| Pm1 | Peanut | male | 21.02 | 54.43 | 63.73 | 75.28 | 70.3 |
| Pm2 | Peanut | male | 9.51 | 3.92 | 8.98 | 29.32 | 18.26 |
| Pm3 | Peanut | male | 53.14 | 23.56 | 34.57 | 34.23 | 38.13 |
| Pm4 | Peanut | male | 17.59 | 29.29 | 33.47 | 16.12 | 76.6 |
| Pm5 | Peanut | male | 47.69 | 3.36 | 26.65 | 27.97 | 34.19 |
| Pm6 | Peanut | male | 30.49 | 37.72 | 25.13 | 16.27 | 24.27 |
| Pm7 | Peanut | male | 14.26 | 4.12 | 3.96 | 25.55 | 89.99 |
| Pm8 | Peanut | male | 6.77 | 12.4 | 5.5 | 6.18 | 28.68 |
| Pm9 | Peanut | male | 9.52 | 4.75 | 9.87 | 3.51 | 10.44 |
| Pm10 | Peanut | male | 8.36 | 7.28 | 3.15 | 45.56 | 29.82 |
| Pf1 | Peanut | female | 9.42 | 40.92 | 29.37 | 32.91 | 239.5 |
| Pf2 | Peanut | female | 4.73 | 39.44 | 48.84 | 80.21 | 8.1 |
| Pf3 | Peanut | female | 43.39 | 20.33 | 18.3 | 31.07 | 34.37 |
| Pf4 | Peanut | female | 22.09 | 86.34 | 52.61 | 36.3 | 26.19 |
| Pf5 | Peanut | female | 47.35 | 40.38 | 45.14 | 67.99 | 18.51 |
| Pf6 | Peanut | female | 10.5 | 35.43 | 2.77 | 13.2 | 38.86 |
| Pf7 | Peanut | female | 6.48 | 20.6 | 34.71 | 9.39 | 8.85 |
| Pf8 | Peanut | female | 17.75 | 19.66 | 30.44 | 20.59 | 10.73 |
| Pf9 | Peanut | female | 39.03 | 26.57 | 6.79 | 26.64 | 13.29 |
| Pf10 | Peanut | female | 11.13 | 28.93 | 46.73 | 5.98 | 16.56 |
| Cm1 | Control | male | 60.2 | 64.4 | 78.65 | 66.78 | 72.44 |
| Cm2 | Control | male | 31.68 | 84.21 | 48.84 | 81.81 | 26.17 |
| Cm3 | Control | male | 189.59 | 7.07 | 57.54 | 15.23 | 30.29 |
| Cm4 | Control | male | 12.37 | 9.94 | 18.13 | 12.72 | 137.39 |
| Cm5 | Control | male | 55.14 | 2.62 | 2.49 | 2.99 | 24.55 |
| Cm6 | Control | male | 3.37 | 28.17 | 39.45 | 10.01 | 49.71 |
| Cm7 | Control | male | 13.63 | 1.64 | 4.51 | 7.32 | 8.98 |
| Cm8 | Control | male | 6.49 | 30.47 | 8.46 | 29.77 | 41.81 |
| Cm9 | Control | male | 68.34 | 26.89 | 21.47 | 13.65 | 28.04 |
| Cm10 | Control | male | 25.35 | 14.15 | 1.53 | 7.75 | 5.04 |
| Cf1 | Control | female | 16.87 | 116.61 | 6.99 | 17.68 | 23.45 |
| Cf2 | Control | female | 23.13 | 22.15 | 28.16 | 4.18 | 11.03 |
| Cf3 | Control | female | 99.55 | 41.98 | 34.18 | 56.65 | 33.94 |
| Cf4 | Control | female | 14.38 | 46.04 | 29.37 | 15.36 | 53.14 |
| Cf6 | Control | female | 8.31 | 29.43 | 12.43 | 12.43 | 7.72 |
| Cf7 | Control | female | 241.96 | 25.95 | 50.72 | 186.01 | 27.87 |
| Cf8 | Control | female | 16.84 | 25.02 | 27.13 | 14.67 | 7.6 |
| Cf9 | Control | female | 1.93 | 46.89 | 8.45 | 0.55 | 6.47 |
| Cf10 | Control | female | 2.59 | 2.03 | 1.46 | 8.74 | 11.01 |

| Cort_Day18 | Cort_Day19 | Cort_Day22 | Latency1 | Latency2 | Latency3 | Latency4 | Errors1 |
|---|---|---|---|---|---|---|---|
| 52.23 | 48.45 | 16.23 | 30.2 | 146.32 | 32.36 | 46.96 | 0 |
| 13.83 | 59.57 | 31.18 | 23.44 | 191.84 | 24.88 | 13.08 | 2 |
| 26.53 | 37.31 | 53.6 | 102.96 | 60.88 | 26.16 | 141.28 | 7 |
| 55.82 | 16.76 | 5.97 | 600 | 600 | 600 | 600 | 0 |
| 55.59 | 19.04 | 16.67 | 95.32 | 229.8 | 106.04 | 31.04 | 0 |
| 272.86 | 57.64 | 28.02 | 48.6 | 40.2 | 13.68 | 22.44 | 2 |
| 28.25 | 50.15 | 35.4 | 124.52 | 42.56 | 100.6 | 122.4 | 9 |
| 27.26 | 58.3 | 18.24 | 143.04 | 65.44 | 42.68 | 270.6 | 7 |
| 4.26 | 1.59 | 6.88 | 600 | 336.84 | 61.52 | 380 | 21 |
| 11.17 | 21.73 | 17.3 | 449.44 | 177.88 | 229.88 | 129.52 | 13 |
| 7.86 | 9.51 | 18.79 | 81.8 | 151.76 | 118.8 | 126.08 | 3 |
| 4.11 | 12.02 | 60.88 | 82.92 | 90.88 | 22.28 | 11.88 | 5 |
| 21.22 | 32.28 | 25.91 | 92.08 | 50.32 | 29.76 | 8.92 | 4 |
| 57.51 | 58.48 | 44.89 | 152.48 | 24.04 | 56.84 | 51.28 | 11 |
| 57.04 | 52.33 | 26.7 | 600 | 600 | 600 | 461.32 | 3 |
| 71.2 | 41.31 | 27.29 | 96.72 | 52.92 | 150.72 | 33.88 | 6 |
| 3.96 | 27.69 | 266.78 | 359.04 | 409.64 | 74.24 | 64.92 | 1 |
| 86.1 | 56.64 | 8.54 | 567.12 | 60 | 353.44 | 228.96 | 6 |
| 10.34 | 7.49 | 19.01 | 600 | 600 | 42.56 | 269.68 | 7 |
| 43.8 | 3.3 | 4.47 | 181.12 | 46.36 | 9.4 | 69.24 | 13 |
| 60.76 | 35.33 | 57.81 | 127.08 | 53.16 | 24.72 | 23.32 | 10 |
| 54.74 | 51.08 | 350.03 | 107.88 | 137.6 | 7.08 | 31.92 | 7 |
| 48.37 | 51.97 | 56.08 | 241.96 | 78.04 | 42.84 | 17.2 | 13 |
| 68.76 | 90.63 | 33.7 | 93.64 | 103.08 | 48.36 | 28.76 | 4 |
| 20.69 | 10.76 | 17.96 | 600 | 600 | 550.88 | 600 | 0 |
| 102.88 | 84.16 | 45.23 | 600 | 118.16 | 178.12 | 74.72 | 16 |
| 88.15 | 92.29 | 105.62 | 37.16 | 45.8 | 63.88 | 35.48 | 1 |
| 49.02 | 81.86 | 21.88 | 86.76 | 143.28 | 13.32 | 29.16 | 5 |
| 34.33 | 3.2 | 9.25 | 148 | 162.8 | 54.36 | 164.24 | 5 |
| 18.87 | 21.13 | 17.92 | 112.16 | 110.04 | 178.56 | 82.48 | 5 |
| 18.48 | 107.51 | 7.45 | 78.2 | 600 | 600 | 90.36 | 1 |
| 2.77 | 0.78 | 18.68 | 84.8 | 62.8 | 310.6 | 132.12 | 7 |
| 21.75 | 15.66 | 5.14 | 174.16 | 75.24 | 60.08 | 468.32 | 7 |
| 28.86 | 37.12 | 7.2 | 83.8 | 67.92 | 600 | 251.96 | 7 |
| 17.74 | 24.21 | 29.14 | 86.56 | 34.88 | 68.44 | 89.28 | 3 |
| 131.19 | 50.72 | 43.15 | 110.24 | 59.4 | 64 | 65.2 | 7 |
| 10.03 | 5.24 | 58.3 | 600 | 600 | 600 | 600 | 10 |
| 40.91 | 81.27 | 17.01 | 122.72 | 61.16 | 29.24 | 65.72 | 3 |
| 3.29 | 6.82 | 14.8 | 144.04 | 157.6 | 26.76 | 71.2 | 10 |
| 19.84 | 16.93 | 21.19 | 153.96 | 59.2 | 24.8 | 54.48 | 12 |
| 71.4 | 60.76 | 62.68 | 20.6 | 20.28 | 7.92 | 11.92 | 0 |
| 24.8 | 63.18 | 34.88 | 112.76 | 189.6 | 22.84 | 45.4 | 4 |
| 71.3 | 84.88 | 55.47 | 278.84 | 155.28 | 60.88 | 17.4 | 11 |
| 69.48 | 73.17 | 24.58 | 114.76 | 65.64 | 67.12 | 97.56 | 4 |
| 41.32 | 42.94 | 51.28 | 143.72 | 40.04 | 32.68 | 18.56 | 3 |
| 102.21 | 58.02 | 81.65 | 159.72 | 102.48 | 19.36 | 33.96 | 12 |
| 79.53 | 125.73 | 47.46 | 88.12 | 94.32 | 29.92 | 34.36 | 4 |
| 27.06 | 45.27 | 5.52 | 297.64 | 92.16 | 89.48 | 79.2 | 21 |
| 11.94 | 3.62 | 17.31 | 91.92 | 287.48 | 64.08 | 70.68 | 6 |
| 36.43 | 26.18 | 27.96 | 600 | 600 | 600 | 181 | 10 |
| 24.61 | 17.69 | 26.18 | 209.36 | 141.48 | 117 | 62.64 | 11 |
| 46.19 | 43.68 | 137.13 | 99.4 | 104.16 | 14.72 | 43.16 | 6 |
| 25.37 | 33.97 | 24.49 | 72.88 | 32.76 | 20.36 | 40.56 | 6 |
| 51.39 | 45.51 | 26.67 | 122.32 | 38.88 | 25.4 | 118.28 | 10 |
| 4.42 | 34.49 | 32.29 | 144.76 | 289.12 | 160.68 | 126.8 | 8 |
| 44.34 | 33.38 | 10.57 | 186.04 | 52.88 | 11.04 | 82.8 | 11 |
| 7.85 | 8.35 | 2.19 | 600 | 171.12 | 53.08 | 55.12 | 10 |
| 17.24 | 28.27 | 1.42 | 163.64 | 158.36 | 180.12 | 113.44 | 13 |
| 14.84 | 15.5 | 14.58 | 129 | 67.48 | 252.16 | 70.4 | 6 |
| 32.38 | 47.1 | 21.19 | 600 | 600 | 600 | 600 | 6 |
| 22.61 | 35.33 | 78.5 | 302.8 | 27.72 | 21.08 | 30.2 | 15 |
| 30.14 | 82.55 | 105.62 | 126.44 | 87.76 | 51.76 | 73.32 | 3 |
| 42.27 | 65.42 | 42.38 | 144.96 | 25.56 | 11.12 | 180.04 | 3 |
| 72.91 | 77.92 | 123.65 | 100.96 | 16.24 | 31.2 | 87.6 | 6 |
| 35.4 | 395.29 | 65.72 | 444.56 | 67.76 | 76.96 | 600 | 16 |
| 27.44 | 9.66 | 20.29 | 233.88 | 29.68 | 37.92 | 43.32 | 18 |
| 8.91 | 28.64 | 2.51 | 175.44 | 261.44 | 42.92 | 136.92 | 8 |
| 55.03 | 25.35 | 16.28 | 106.88 | 73 | 34.32 | 100 | 6 |
| 30.77 | 34.41 | 21.28 | 115.08 | 18.4 | 13.16 | 16 | 10 |
| 16.93 | 12.34 | 6.28 | 126.92 | 112.8 | 127.96 | 100.32 | 6 |
| 11.55 | 34.66 | 5.4 | 34.04 | 54.68 | 600 | 49.56 | 0 |
| 16.85 | 20.07 | 75.54 | 46.88 | 79.32 | 204.52 | 37.56 | 4 |
| 72.05 | 70.94 | 85.65 | 200.84 | 57.8 | 600 | 600 | 6 |
| 118.26 | 85.49 | 90.62 | 80.32 | 19.92 | 23.12 | 56.32 | 4 |
| 65.51 | 31.08 | 21.1 | 600 | 600 | 600 | 600 | 0 |
| 50.48 | 150.2 | 3 | 401.24 | 144.76 | 89.52 | 100.24 | 15 |
| 10.65 | 44.94 | 1.63 | 251.4 | 189.8 | 164.92 | 175.44 | 6 |
| 4.05 | 7.29 | 12.86 | 92.44 | 333.84 | 78.68 | 51.2 | 4 |
| 13.27 | 1.16 | 14.18 | 494.48 | 150.56 | 102.36 | 61 | 15 |

| Errors2 | Errors3 | Errors4 | Movement1 | Movement2 | Movement3 | Movement4 | C13:0 |
|---|---|---|---|---|---|---|---|
| 2 | 3 | 6 | 29.93 | 17.33 | 50.56 | 55.71 | 0 |
| 4 | 0 | 0 | 65.53 | 22.89 | 46.3 | 52.91 | 0 |
| 5 | 3 | 14 | 55.48 | 59.2 | 65.14 | 54.93 | 0 |
| 0 | 1 | 1 | 0.94 | 0 | 3.46 | 0.71 | 0 |
| 6 | 5 | 0 | 20.56 | 29.35 | 41.27 | 46.01 | 0 |
| 2 | 0 | 2 | 45.84 | 40.9 | 55.85 | 52.58 | |
| 1 | 10 | 9 | 52.3 | 33.08 | 51.77 | 48.73 | 0.210351247 |
| 3 | 6 | 7 | 40.02 | 38.88 | 70.76 | 13.51 | 0.604353866 |
| 17 | 3 | 8 | 28.06 | 33.42 | 33.55 | 14.92 | 0.36324994 |
| 9 | 2 | 2 | 23.22 | 42.32 | 11.17 | 16.09 | 0.142906621 |
| 6 | 4 | 6 | 39.31 | 29.73 | 28.32 | 46.92 | 0 |
| 6 | 0 | 0 | 31.55 | 39.48 | 58.53 | 59.93 | 0 |
| 4 | 2 | 0 | 40.27 | 53.02 | 61.16 | 77.58 | 0 |
| 1 | 3 | 3 | 41.74 | 58.57 | 50.81 | 40.48 | 0 |
| 1 | 1 | 7 | 11.83 | 1.81 | 1.92 | 18.62 | 0 |
| 4 | 13 | 4 | 45.04 | 63.64 | 45.3 | 70.37 | 0.06628352 |
| 9 | 4 | 3 | 9.64 | 17.23 | 42.94 | 48.68 | 0.193568759 |
| 3 | 0 | 1 | 7.57 | 27.53 | 2.42 | 7.02 | 0.794567215 |
| 3 | 0 | 4 | 10.88 | 3.87 | 40.32 | 12.43 | 0.864474492 |
| 2 | 1 | 5 | 45.87 | 42.8 | 68.09 | 29.29 | 0.083704966 |
| 4 | 5 | 4 | 35.06 | 44.32 | 81.88 | 72.21 | 0 |
| 10 | 0 | 4 | 34.07 | 40.12 | 71.75 | 61.03 | 0 |
| 6 | 6 | 3 | 35.53 | 47.82 | 61.81 | 64.42 | |
| 3 | 2 | 2 | 33.87 | 29.3 | 21.42 | 32.96 | 0 |
| 0 | 0 | 5 | 1.63 | 0 | 3.61 | 10.61 | 0 |
| 6 | 15 | 7 | 14.67 | 40.96 | 52.64 | 43.68 | |
| 4 | 11 | 9 | 48.55 | 46.64 | 53.66 | 63.81 | 0.156494113 |
| 12 | 1 | 6 | 41.68 | 39.45 | 56.46 | 62.69 | 0.537517279 |
| 5 | 4 | 8 | 40.62 | 23.69 | 57.17 | 33.41 | 0.260744448 |
| 4 | 9 | 10 | 29.49 | 26.9 | 34.36 | 45.93 | 0.226468264 |
| 5 | 2 | 3 | 25.32 | 4.31 | 1.6 | 33.47 | 0 |
| 5 | 4 | 3 | 46.18 | 38.22 | 7.34 | 19.68 | 0.123228589 |
| 5 | 2 | 9 | 40.06 | 60.87 | 34.35 | 12.17 | 0 |
| 5 | 6 | 5 | 53.46 | 44.05 | 6.62 | 16.4 | 0.070943549 |
| 2 | 3 | 5 | 37.2 | 40.25 | 32.85 | 34.27 | 0 |
| 4 | 4 | 3 | 41 | 34.41 | 43.19 | 38.22 | 0.172314319 |
| 13 | 6 | 5 | 15.13 | 12.29 | 5.06 | 4.34 | 0.256546113 |
| 5 | 1 | 3 | 19.69 | 45.45 | 28.73 | 26.9 | 0.977249795 |
| 9 | 3 | 4 | 47.6 | 30.99 | 56.2 | 38.2 | 0.154935939 |
| 6 | 3 | 5 | 47.03 | 52.16 | 54.84 | 40.31 | 0.339362313 |
| 1 | 0 | 0 | 63.88 | 60.16 | 88.89 | 77.18 | 0 |
| 8 | 0 | 3 | 27.03 | 27.34 | 38 | 51.71 | 0 |
| 13 | 5 | 0 | 24.95 | 41.4 | 43.1 | 33.8 | 0 |
| 2 | 6 | 8 | 35.59 | 33.94 | 62.04 | 39.77 | 0.06091989 |
| 4 | 2 | 3 | 24.52 | 67.33 | 47.74 | 63.79 | 0 |
| 7 | 1 | 4 | 39.27 | 45.67 | 66.74 | 61.37 | 0.112807713 |
| 7 | 3 | 5 | 62.14 | 42.58 | 47.33 | 54.02 | 0.372292948 |
| 3 | 2 | 3 | 42.92 | 31.51 | 30.26 | 30 | 0.052846673 |
| 13 | 5 | 6 | 52.79 | 26.02 | 43.7 | 43.29 | 0.08233183 |
| 6 | 5 | 10 | 12.09 | 4.54 | 4.99 | 49.79 | 0.090890089 |
| 5 | 4 | 6 | 25.75 | 24.88 | 23.32 | 35.31 | 0 |
| 9 | 0 | 3 | 44.63 | 46.12 | 63.59 | 51.81 | 0 |
| 0 | 0 | 2 | 59 | 49.08 | 65.42 | 47.93 | 0.071341215 |
| 2 | 4 | 12 | 50.39 | 60.29 | 74.49 | 50.39 | 0.082372323 |
| 7 | 6 | 4 | 52.31 | 27.49 | 40.38 | 29.53 | 0.093331951 |
| 5 | 0 | 5 | 38.21 | 53.03 | 47.83 | 42.22 | |
| 4 | 4 | 4 | 10.01 | 14.98 | 34.89 | 34.03 | 0.361681612 |
| 6 | 5 | 0 | 36.01 | 20.31 | 12.28 | 4.41 | 0.753813399 |
| 6 | 6 | 8 | 41.33 | 49.73 | 16.39 | 47.22 | 0.298283508 |
| 9 | 2 | 4 | 9.49 | 12.42 | 2.55 | 3.93 | 0.213134434 |
| 3 | 3 | 4 | 33.91 | 78.35 | 84.44 | 72.32 | 0 |
| 4 | 4 | 7 | 23.09 | 35.32 | 63.83 | 49.37 | 0.143884892 |
| 1 | 0 | 6 | 21.22 | 37.72 | 46.4 | 21.57 | 0 |
| 0 | 3 | 3 | 37.48 | 46.8 | 61.28 | 29.59 | 0 |
| 3 | 4 | 13 | 28.84 | 47.99 | 53.74 | 13.13 | 0.175185491 |
| 1 | 3 | 3 | 43.06 | 48.65 | 66.14 | 55.12 | 0.481041306 |
| 8 | 3 | 2 | 48.4 | 21.79 | 39.98 | 20.42 | 0.442283092 |
| 3 | 4 | 4 | 38.32 | 39.51 | 64.8 | 21.6 | 0.937086697 |
| 1 | 2 | 3 | 39.28 | 61.74 | 62.61 | 62 | 0.206967842 |
| 6 | 6 | 8 | 51.91 | 47.02 | 31.1 | 47.33 | 0.122154546 |
| 6 | 4 | 2 | 26.44 | 59.33 | 2.45 | 28.33 | 0.204834084 |
| 5 | 9 | 4 | 51.79 | 36.16 | 29.63 | 60.17 | 0.365497076 |
| 7 | 9 | 3 | 31.97 | 69.34 | 8.72 | 2.61 | 0.176056338 |
| 2 | 4 | 7 | 37.7 | 72.29 | 73.88 | 61.36 | 0.11216478 |
| 0 | 0 | 0 | 0.87 | 0.03 | 0 | 0 | 0.071144143 |
| 10 | 6 | 6 | 26.2 | 39.71 | 35.84 | 37.39 | 0.288802729 |
| 1 | 5 | 5 | 13.75 | 6.43 | 15.38 | 11.24 | 0.847443624 |
| 12 | 6 | 5 | 42.67 | 22.91 | 44.23 | 60.31 | 0.260773197 |
| 6 | 7 | 6 | 21.19 | 24.81 | 33.06 | 41.64 | 0.245096507 |

| C14:0 | C14:1 | C15:0 | C16:0 | C16:1n7c | C17:0 | C17:1 | C18:0 |
|---|---|---|---|---|---|---|---|
| 0.357781753 | 0.168367884 | 0.557718615 | 15.91076502 | 0.526149637 | 0.88393139 | 0 | 12.57497632 |
| 0.348682186 | 0.123064301 | 0.420469695 | 13.12685878 | 0.430725054 | 0.656342939 | 0 | 9.834888729 |
| 0.441826215 | 0.262991795 | 0.58910162 | 11.88722912 | 0.273511466 | 0.894172102 | 0 | 11.00357669 |
| 0.437773609 | 0.239733167 | 0.521159058 | 15.29080675 | 0.896393579 | 0.583698145 | 0 | 13.41463415 |
| 0.480217142 | 0.323624595 | 0.636809688 | 15.23123499 | 0.741204719 | 1.002192296 | 0 | 12.07850506 |
| 0.442268873 | 0.168533104 | 1.545461588 | 16.79711054 | 0.505495308 | 1.976984712 | 0.178018318 | 13.96743427 |
| 0.729038739 | 0.195938061 | 3.256895001 | 16.30073207 | 0.142885634 | 1.724267704 | 0.141684041 | 12.01191484 |
| 0.726001059 | 0.368071875 | 2.300373012 | 13.82343796 | 0.565259805 | 2.09451618 | 0.173821446 | 11.30521374 |
| 0.621067045 | 0.226815908 | 1.739351769 | 13.55879548 | 0.445219709 | 1.708901445 | 0.050622759 | 12.1535314 |
| 0.382323176 | 0.112447993 | 0.46103677 | 15.5965366 | 0.764646351 | 0.663443158 | 0.247385584 | 10.41268413 |
| 0.205973223 | 0.123583934 | 0.288362513 | 13.55303811 | 0.432543769 | 0.535530381 | 0 | 10.65911432 |
| 0.438825619 | 0.114930519 | 0.42837739 | 15.06634625 | 0.867203009 | 0.689583116 | 0 | 10.56315954 |
| 0.42646141 | 0.124817974 | 0.416059913 | 14.7701269 | 0.790513834 | 0.728104847 | 0 | 9.309340545 |
| 0.305778153 | 0.07380852 | 0.390130747 | 15.32053986 | 0.738085196 | 0.832981864 | 0 | 10.64951497 |
| 0.43701671 | 0.182155825 | 1.147406298 | 14.82846111 | 0.541017811 | 1.822407852 | 0.14731592 | 13.66386044 |
| 1.054938145 | 0.403153481 | 1.78399175 | 17.4821744 | 1.026184601 | 1.38677559 | 0.202079309 | 11.3259122 |
| 0.74059619 | 0.39903899 | 3.848062124 | 13.46039842 | 0.38891336 | 1.989080027 | 0.196173821 | 13.25754445 |
| 0.827336335 | 0.42983326 | 2.802219455 | 15.1369577 | 0.536123887 | 2.063961362 | 0.169620195 | 12.32520515 |
| 0.41889039 | 0.153629132 | 1.522178968 | 12.8017079 | 0.256921366 | 1.426623025 | 0.029584936 | 14.10608053 |
| 0.159151194 | 0.148541114 | 0.562334218 | 12.79575597 | 0.381962865 | 1.092838196 | 0 | 13.82493369 |
| 0.103348491 | 0.072343944 | 0.248036379 | 9.88011575 | 0.248036379 | 0.423728814 | 0.144687888 | 9.022323274 |
| 0.568527919 | 0.081218274 | 0.355329949 | 16.12182741 | 1.197969543 | 0.578680203 | 0.14213198 | 8.040609137 |
| 0.133456524 | 0.14372241 | 0.379837799 | 13.1916641 | 0.687814393 | 0.872600349 | 0.164254183 | 12.07268248 |
| 0.718106492 | 0.238358052 | 1.438204097 | 17.00032613 | 0.439207691 | 1.496281248 | 0.131188112 | 12.55447092 |
| 0.808492363 | 0.244468985 | 3.256751066 | 13.02016978 | 0.30232194 | 2.12745387 | 0.120248816 | 11.75559703 |
| 1.053727918 | 0.438357938 | 1.962346288 | 15.97756066 | 0.695912627 | 1.492901671 | 0.196126099 | 12.07181799 |
| 0.870080685 | 0.294891297 | 2.635674775 | 1.790977479 | 0.429082421 | 1.143826632 | 0.096766322 | 13.61928693 |
| 0.414057766 | 0.161583518 | 0.424156736 | 15.31003838 | 0.777620683 | 0.616037164 | 0.100989699 | 8.281155322 |
| 0.45183816 | 0.11295954 | 0.472376258 | 13.23680427 | 0.605873896 | 0.759909632 | 0.11295954 | 8.975148901 |
| 0.182463254 | 0.111505322 | 0.385200203 | 11.84997466 | 0.385200203 | 0.881905727 | 0.111505322 | 11.44450076 |
| 0.374987332 | 0.22296544 | 0.648626736 | 11.76649438 | 0.344582953 | 0.668896321 | 0.070943549 | 9.526705179 |
| 0.162651215 | 0.081325607 | 0.284639626 | 14.41496391 | 0.782758971 | 0.66077056 | 0.193148318 | 7.268476161 |
| 0.649689094 | 0.196492914 | 1.296503679 | 14.83878861 | 0.574548404 | 1.465683186 | 0.051635169 | 11.80128669 |
| 0.658082701 | 0.230534718 | 1.638036158 | 17.51844253 | 0.522351276 | 1.366078783 | 0.197228398 | 13.84981607 |
| 1.138431238 | 0.461648768 | 3.644929962 | 13.79013337 | 0.643351365 | 2.761373051 | 0.159047101 | 11.74207003 |
| 0.415384896 | 0.13043223 | 1.452856218 | 12.31161338 | 0.247937413 | 2.025946159 | 0.071775313 | 9.988342423 |
| 0.493417003 | 0.184969333 | 2.165419766 | 13.07656406 | 0.359437837 | 1.438256007 | 0.120264589 | 12.3533849 |
| 0.336528656 | 0.132571895 | 0.438507037 | 13.38976137 | 0.305935142 | 0.713848664 | 0.214154599 | 10.45278401 |
| 0.202839757 | 0.14198783 | 0.365111562 | 11.01419878 | 0.172413793 | 0.618661258 | 0.111561866 | 10.50709939 |
| 0.222536921 | 0.13149909 | 0.4855351 | 11.15719199 | 0.202306292 | 0.799109852 | 0.101153146 | 10.4693506 |
| 0.324906082 | 0.142146411 | 0.426439232 | 13.07746979 | 0.629505534 | 0.761498629 | 0.213219616 | 11.96060514 |
| 0.355871886 | 0.193187595 | 0.538891713 | 12.98423996 | 0.4473818 | 0.843924759 | 0.2236909 | 12.7808846 |
| 0.459583722 | 0.139687221 | 1.338648879 | 14.91732743 | 0.313500669 | 1.678643321 | 0.105841788 | 13.12077958 |
| 0.774295093 | 0.285541819 | 2.928869347 | 18.25589755 | 0.695173296 | 1.389151513 | 0.156151348 | 12.33896304 |
| 0.296528705 | 0.130856719 | 0.986770226 | 12.66757677 | 0.301419513 | 1.12960004 | 0.140492052 | 11.96741287 |
| 0.324791859 | 0.17056936 | 1.336084655 | 12.23902527 | 0.315884116 | 1.436740711 | 0.090390074 | 10.3645693 |
| 0.423506752 | 0.137368237 | 1.18135036 | 13.01686385 | 0.278186745 | 0.913095914 | 0.063251672 | 11.6854646 |
| 0.332761924 | 0.121004336 | 0.373096703 | 13.48189977 | 0.484017344 | 0.494101039 | 0.141171725 | 10.22486639 |
| 0.317622951 | 0.112704918 | 0.389344262 | 13.97540984 | 0.727459016 | 0.717213115 | 0.204918033 | 11.42418033 |
| 0.305748064 | 0.132490828 | 0.377089278 | 13.0044843 | 0.509580106 | 0.570729719 | 0.19364044 | 11.80187525 |
| 0.27800659 | 0.102965404 | 0.391268534 | 12.36614498 | 0.339785832 | 0.545716639 | 0 | 11.4291598 |
| 0.497770403 | 0.145183034 | 0.487400187 | 15.44125272 | 0.798506689 | 0.632583221 | 0.311106502 | 7.829513637 |
| 0.701699183 | 0.172758702 | 1.30701353 | 16.60719344 | 0.782091358 | 5.43004823 | 0.157966854 | 12.05682482 |
| 0.750189451 | 0.262794133 | 2.58659728 | 13.02627529 | 0.516410782 | 2.683166237 | 0.152486413 | 12.44360298 |
| 0.58761683 | 0.29010036 | 1.904359682 | 13.15171789 | 0.378805765 | 1.660987743 | 0.130440719 | 10.63080615 |
| 0.536334487 | 0.207909717 | 2.707079991 | 13.07750488 | 0.448482087 | 1.448374487 | 0.132667913 | 12.92727302 |
| 0.525394046 | 0 | 0.752034614 | 15.21582363 | 0.329659009 | 0.659318018 | 0 | 9.807355517 |
| 0.503597122 | 0.154162384 | 0.431654676 | 14.43987667 | 0.441932169 | 0.637204522 | 0.22610483 | 9.362795478 |
| 0 | 0 | 0 | 4.860073698 | 0 | 0.527835873 | 0 | 22.25873917 |
| 0 | 0.050362611 | 0.161160355 | 8.632151491 | 0.171232877 | 0.916599517 | 0 | 14.19218372 |
| 0.5358615 | 0.195795548 | 0.5358615 | 16.43652102 | 0.89653751 | 1.34995878 | 0.391591096 | 11.94352844 |
| 0.848688001 | 0.209244545 | 2.595946401 | 17.6609787 | 0.686812213 | 1.957655476 | 0.118119721 | 13.26847178 |
| 1.290846634 | 0.418449423 | 2.957471664 | 18.18181818 | 0.514086 | 2.213830674 | 0.145626238 | 14.91650934 |
| 0.935730031 | 0.408758288 | 3.759832582 | 15.33582169 | 0.594351912 | 1.745340133 | 0.113544355 | 11.11150186 |
| 0.540364611 | 0.41759315 | 1.785285733 | 13.90306178 | 0.333148957 | 2.174165529 | 0.130689503 | 10.93389133 |
| 0.562710692 | 0.1996938 | 1.681953867 | 14.0904305 | 0.265612832 | 2.528188763 | 0.091175655 | 13.31190076 |
| 0.604260549 | 0.286767718 | 0.727161 | 14.95288816 | 0.757886112 | 0.65546907 | 0.235559197 | 9.463334699 |
| 0.584795322 | 0.167084378 | 0.459482038 | 15.44486216 | 0.824979114 | 0.626566416 | 0.250626566 | 10.68295739 |
| 0.621375311 | 0.176056338 | 0.569594035 | 14.75766363 | 0.57995029 | 0.869925435 | 0 | 8.688898094 |
| 0.50983991 | 0.214132762 | 0.50983991 | 15.47873968 | 0.723972673 | 0.815743856 | 0.28551035 | 10.74742531 |
| 0.577896995 | 0.288415869 | 0.981857275 | 17.67499291 | 0.765422759 | 1.50402667 | 0.148764171 | 13.01000329 |
| 0.661570258 | 0.270484693 | 1.867787562 | 18.72931454 | 0.72759906 | 1.493068129 | 0.150652287 | 12.14597755 |
| 0.83627728 | 0.378427514 | 4.028860786 | 14.28799561 | 0.303642082 | 3.106785995 | 0.163585158 | 12.59762059 |
| 1.0538441 | 0.438406271 | 1.962562651 | 15.9793223 | 0.695989357 | 1.493066274 | 0.196147723 | 12.06212327 |
| 1.116772568 | 0.42581294 | 2.723006993 | 15.59669869 | 0.655160883 | 1.670448259 | 0.155723219 | 12.14355559 |

| C18:1n9c | C18:1n7c | C18:2n6t | C18:2n6c | C18:3n6 | C20:0 | C18:3n3 | C20:1n9 |
|---|---|---|---|---|---|---|---|
| 13.49047669 | 0 | 0.368304746 | 45.7960644 | 0 | 0 | 5.84026097 | 0.220982848 |
| 10.49123167 | 1.415239463 | 0.287150036 | 49.22572044 | 0 | 0.256383961 | 9.455440468 | 0.184596452 |
| 11.7083947 | 0 | 0.231432779 | 46.42331159 | 0 | 0 | 14.14895855 | 0 |
| 14.6549927 | 0 | 0.239733167 | 39.05565979 | 0 | 0.114654993 | 10.92349385 | 0.229309985 |
| 13.40432195 | 0 | 0.386261614 | 41.54922226 | 0 | 0.125274037 | 10.72136966 | 0.187911055 |
| | | | | | | | |
| 13.99266993 | 1.108172451 | 0.135884001 | 39.08772383 | 0.156605865 | 0.076476613 | 5.330885427 | 0.021183643 |
| 12.16260445 | 1.009996342 | 0.090232522 | 35.77265037 | 0.114966375 | 0.053728893 | 11.92822088 | 0.047148394 |
| 10.3766914 | 1.035547136 | 0.095196692 | 39.9113172 | 0.114633072 | 0.114731325 | 13.23411131 | 0.078297245 |
| 11.57270467 | 1.017687941 | 0.058351281 | 40.07613787 | 0.071028416 | 0.082429528 | 12.55875205 | 0.0445402 |
| 13.76363432 | 0 | 0.337343978 | 46.9245474 | 0 | 0.146182391 | 6.904306758 | 0.112447993 |
| 12.87332647 | 0 | 0.216271885 | 49.52626159 | 0 | 0.308959835 | 8.383110196 | 0.195674562 |
| 13.7498694 | 0 | 0.208964581 | 46.49461916 | 0 | 0.156723435 | 7.940654059 | 0.125378748 |
| 11.82650302 | 0 | 0.228832952 | 48.72061577 | 0 | 0.176825463 | 9.746203453 | 0.124817974 |
| 15.49978912 | 0 | 0.137072965 | 45.57148882 | 0.168705188 | 0 | 4.997891185 | 0.115984817 |
| 11.18170798 | 1.013433096 | 0.115570834 | 40.25480776 | 0.173379441 | 0.013850791 | 9.002841478 | 0.22656901 |
| 14.1628065 | 1.038598823 | 0.108624933 | 38.09815592 | 0.143465791 | 0.111170891 | 7.547481041 | 0.023599295 |
| 10.71742346 | 0.933356248 | 0.348463073 | 39.24545327 | 0.14060411 | 0.09989266 | 8.76365442 | 0.102752386 |
| 1.229895937 | 0.780486436 | 0.127233917 | 49.55585286 | 0.155763292 | 0.146859352 | 8.586049853 | 0.117773319 |
| 12.5631326 | 0.941022005 | 0.071794479 | 38.45325873 | 0.20935593 | 0.131933512 | 11.72681406 | 0.01906018 |
| 10.85411141 | 1.326259947 | 0 | 48.77453581 | 0.106100796 | 0.233421751 | 5.432360743 | 0.084880637 |
| 13.84869781 | 0.992145515 | 0.144687888 | 55.15708971 | 0.134353038 | 0.155022737 | 6.97602315 | 0.175692435 |
| | | | | | | | |
| 15.02538071 | 0.994923858 | 0.14213198 | 48.26395939 | 0.131979695 | 0.14213198 | 4.964467005 | 0.152284264 |
| 12.49358382 | 1.067652192 | 0.277178934 | 48.32152756 | 0.133456524 | 0.236115389 | 5.779694077 | 0.164254183 |
| | | | | | | | |
| 12.34757184 | 1.175928342 | 0.156344932 | 40.51438709 | 0.148538798 | 0.137639437 | 5.8417162 | 0.102390053 |
| 10.40967593 | 0.909171531 | 0.084321487 | 44.48463979 | 0.161918507 | 0.048952294 | 7.580658383 | 0.039820865 |
| 11.88952766 | 1.043617214 | 0.069543857 | 42.28327037 | 0.109969524 | 0.151410671 | 6.15439557 | 0.440974423 |
| 12.22153671 | 1.188835058 | 0.099663437 | 52.68974453 | 0.11682189 | 0.126885552 | 8.161409831 | 0.019867211 |
| 16.57240961 | 1.07049081 | 0.141385579 | 47.29347607 | 0.131286609 | 0.131286609 | 6.38254898 | 0.141385579 |
| 11.22407065 | 1.037173958 | 0.174573834 | 56.14089135 | 0.123228589 | 0.123228589 | 3.296364757 | 0.11295954 |
| 11.63710086 | 1.003547897 | 0.192600101 | 51.81956412 | 0.141915864 | 0.152052712 | 6.842372022 | 0.111505322 |
| 11.81716834 | 0.841187798 | 0.22296544 | 52.2347218 | 0.091213135 | 0.273639404 | 7.205837641 | 0.233100233 |
| 19.8332825 | 1.108061401 | 0.172816916 | 47.05702958 | 0.142319813 | 0.162651215 | 4.361085697 | 0.142319813 |
| 11.6606496 | 0.936373891 | 0.081521876 | 44.56724239 | 0.144920961 | 0.010373145 | 7.458620374 | 0.008305581 |
| 12.26420782 | 1.021711384 | 0.098250989 | 39.48275489 | 0.206132621 | 0.06639192 | 5.784822454 | 0.004936857 |
| 11.23368243 | 0.955554605 | 0.105598002 | 41.28983983 | 0.156173606 | 0.061566428 | 6.564480348 | 0.092472835 |
| 11.35425749 | 0.984746249 | 0.091785036 | 50.10037664 | 0.137558643 | 0.091339464 | 6.790785993 | 0.201589756 |
| 11.30794922 | 0.970404796 | 0.112827772 | 46.89142158 | 0.113637448 | 0.074828465 | 5.627332948 | 0.10078523 |
| 17.09157659 | 0.805629207 | 0.234550275 | 48.34795023 | 0.091780543 | 0.275341628 | 3.161329798 | 0.193758923 |
| 19.35091278 | 1.064908722 | 0.212981744 | 47.94117647 | 0 | 0.446247465 | 4.929006085 | 0.294117647 |
| 20.24074449 | 0.839571111 | 0.202306292 | 47.20817317 | 0.11126846 | 0.323690067 | 4.65304471 | 0.404612583 |
| 17.04741598 | 1.076251396 | 0.203066301 | 46.33972992 | 0 | 0.233526246 | 4.030866078 | 0.172606356 |
| 15.72953737 | 1.159125572 | 0.162684291 | 44.73817997 | 0 | 0.172852059 | 5.043213015 | 0.152516523 |
| 17.80366818 | 0.894758343 | 0.109361622 | 38.44827096 | 0.200363947 | 0.065401983 | 4.156725829 | 0.294951341 |
| 13.13804319 | 1.022940116 | 0.085501261 | 38.73031808 | 0.199044087 | 0.088295487 | 4.577178474 | 0.045183593 |
| 22.06976627 | 0.885197635 | 0.083016776 | 41.60935354 | 0.146862247 | 0.207323897 | 2.938119878 | 0.352019505 |
| 20.70482468 | 1.02550391 | 0.122906215 | 43.78452995 | 0.102328657 | 0.172613917 | 3.79938515 | 0.2477562 |
| 20.95667544 | 0.957863756 | 0.14542546 | 41.58992505 | 0.060813085 | 0.181628589 | 4.671419692 | 0.202261012 |
| 18.49349602 | 0.826862963 | 0.211757588 | 47.61520621 | 0.090753252 | 0.292427145 | 3.15619643 | 0.262176061 |
| 13.27868852 | 0.901639344 | 0.194672131 | 50.19467213 | 0.112704918 | 0 | 2.612704918 | 0.194672131 |
| 16.57154505 | 0.845902976 | 0 | 46.96290257 | 0.183448838 | 0.214023645 | 3.332653893 | 0.254790053 |
| 12.77800659 | 0.978171334 | 0.247116969 | 51.00906096 | 0 | 0.247116969 | 4.901153213 | 0.175041186 |
| 17.09011718 | 1.04739189 | 0.238514985 | 43.17121228 | 0.114072384 | 0.311106502 | 5.382142487 | 0.248885202 |
| | | | | | | | |
| 12.72114801 | 0.996566659 | 0.1226494 | 38.15271207 | 0.13597272 | 0.063479408 | 4.866939125 | 0.095012478 |
| 16.3401207 | 0.81818355 | 0.174374581 | 41.18840893 | 0.178851082 | 0.110193091 | 2.999925551 | 0.14039186 |
| 20.29878534 | 1.083504231 | 0.112069615 | 40.7278664 | 0.126779948 | 0.184450754 | 3.692156793 | 0.45505362 |
| 14.36813085 | 1.02230833 | 0.125425894 | 42.46358335 | 0.131683783 | 0.083366598 | 4.512817901 | 0.14667787 |
| 12.52704234 | 0.999278871 | 0.236942413 | 48.16112084 | 0 | 0.216338725 | 5.171525703 | 0.288451633 |
| 14.16238438 | 1.130524152 | 0.287769784 | 49.76361768 | 0.102774923 | 0.277492292 | 4.964028777 | 0 |
| 14.44079275 | 0.627427547 | 0 | 45.50343591 | 0 | 0.667264217 | 6.124887959 | 0.527835873 |
| 14.68573731 | 1.369863014 | 0.322320709 | 48.37832393 | 0 | 0.352538276 | 7.020547945 | 0.241740532 |
| 12.58244023 | 1.154163232 | 0.247320692 | 43.36356142 | 0.133965375 | 0.185490519 | 4.85366859 | 0.185490519 |
| 9.856086849 | 0.948482212 | 0.054973857 | 41.23768781 | 0.070392636 | 0.057198697 | 3.024508772 | 0.520727414 |
| 11.66838237 | 1.019665663 | 0.145649461 | 35.40676963 | 0.197692719 | 0.099790218 | 5.269798691 | 0.126453803 |
| 11.75022205 | 0.991151797 | 0.188853237 | 41.98570833 | 0.145246701 | 0.084202275 | 6.067912632 | 0.081825329 |
| 12.53081304 | 1.067075674 | 0.127733673 | 46.53938841 | 0.042074305 | 0.17555244 | 6.135008121 | 0.172826508 |
| 11.10194628 | 1.045489486 | 0.076543713 | 45.44312677 | 0.127161532 | 0.095663955 | 5.152802776 | 0.059732456 |
| 12.59729619 | 1.085620647 | 0.297009422 | 49.77468251 | 0.143383859 | 0.163867268 | 4.414174519 | 0.174108972 |
| 12.32247285 | 1.117376775 | 0.208855472 | 47.0342523 | 0 | 0.187969925 | 4.166666667 | 0.177527151 |
| 10.82228666 | 0.994200497 | 0.258906379 | 51.73985087 | 0.27961889 | 0.196768848 | 3.769676885 | 0.144987572 |
| 13.74528398 | 1.091057408 | 0.316100744 | 45.53890079 | 0.142755175 | 0.214132762 | 6.322014887 | 0.152951973 |
| 13.93784487 | 1.004749357 | 0.127087294 | 39.02213447 | 0.065855347 | 0.006154121 | 4.994216375 | 0.096469759 |
| 12.79484397 | 0.993795712 | 0.075983731 | 39.93847134 | 0.205090425 | 0.045352673 | 4.75520447 | 0.028525461 |
| 11.74140171 | 0.985260752 | 0.104739356 | 41.9485454 | 0.150977039 | 0.054891302 | 3.739120268 | 0.0864739 |
| 11.89083857 | 1.043732281 | 0.069551525 | 42.28793241 | 0.109981649 | 0.151427365 | 6.155074137 | 0.441023044 |
| 12.01123675 | 0.98024774 | 0.193141691 | 40.58585896 | 0.113117504 | 0.106672435 | 7.274940912 | 0.112177445 |

| C20:2n6 | C20:4n6 | C20:5n3 | C24:0 | C22:5n3 | C22:6n3 | total_n9 | total_n6 |
|---|---|---|---|---|---|---|---|
| 0.34725876 | 2.409765337 | 0 | 0 | 0.368304746 | 0.178890877 | 13.71145954 | 48.92139324 |
| 0.184596452 | 2.902266434 | 0 | 0 | 0.461491129 | 0.19485181 | 10.67582812 | 52.59973336 |
| 0.284031138 | 1.693667158 | 0 | 0.157795077 | 0 | 0 | 11.7083947 | 48.63244267 |
| 0.281425891 | 2.053366688 | 0.135501355 | 0.239733167 | 0.375234522 | 0.312695435 | 14.88430269 | 41.63018553 |
| 0.240108571 | 2.150537634 | 0.062637018 | 0.062637018 | 0.323624595 | 0.292306086 | 13.59223301 | 44.32613008 |
| | | | | | | | |
| 0.356818749 | 2.276367999 | 0.066438095 | 0.161019817 | 0.30611446 | 0.346888397 | 14.1531406 | 42.1367831 |
| 0.340521242 | 2.215117528 | 0.071716321 | 0.075470615 | 0.317432058 | 0.167375157 | 12.34683838 | 38.63390948 |
| 0.320699006 | 1.858775441 | 0.14476387 | 0.091266588 | 0.308979235 | 0.130189734 | 10.5012757 | 42.40177373 |
| 0.33758067 | 2.383521279 | 0.051059716 | 0.035846618 | 0.349987052 | 0.141349122 | 11.68880943 | 43.1033783 |
| 0.224895986 | 2.552569437 | 0 | 0.056223996 | 0.337343978 | 0 | 13.87608231 | 50.0393568 |
| 0.339855819 | 1.894953656 | 0 | 0.144181256 | 0.226570546 | 0.092687951 | 13.06900103 | 51.97734295 |
| 0.261205726 | 2.131438721 | 0 | 0.052241145 | 0.376136245 | 0.334343329 | 13.87524815 | 49.09622819 |
| 0.218431454 | 1.986686083 | 0 | 0.072810485 | 0.291241939 | 0 | 11.95132099 | 51.15456626 |
| 0.253057782 | 3.078869675 | 0.105440742 | 0 | 0.811893716 | 0.948966681 | 15.61577394 | 49.20919443 |
| 0.399235883 | 2.830363878 | 0.237582181 | 0.156553997 | 0.566027239 | 0.405773372 | 11.45220108 | 43.97173191 |
| 0.250175899 | 1.884522992 | 0.136673323 | 0.065051461 | 0.259837682 | 0.320930928 | 14.33455054 | 40.60034541 |
| 0.508471531 | 2.784419536 | 0.103838075 | 0.026907164 | 0.374426322 | 0.234840745 | 10.85866368 | 43.2632167 |
| 0.354446219 | 2.351194351 | 0.084958971 | 0.08351166 | 0.394709428 | 0.243404579 | 1.420037398 | 52.69006321 |
| 0.218375935 | 3.313504342 | 0.100087199 | 0.093507101 | 0.619481357 | 0.267063558 | 12.6129339 | 42.29876484 |
| 0.190981432 | 3.129973475 | 0 | 0 | 0.286472149 | 0.519893899 | 11.03448276 | 52.20159151 |
| 0.206696982 | 1.674245556 | 0 | 0 | 0.217031831 | 0.175692435 | 14.02439024 | 57.31707317 |
| | | | | | | | |
| 0.203045685 | 2.538071066 | 0 | 0 | 0.223350254 | 0.131979695 | 15.17766497 | 51.27918782 |
| 0.256647161 | 2.381685659 | 0 | 0 | 0.513294323 | 0.266913048 | 13.11980289 | 51.37049584 |
| | | | | | | | |
| 0.317649377 | 3.107964802 | 0.133495854 | 0.065365717 | 0.384223766 | 0.441962976 | 12.60635858 | 44.38121225 |
| 0.25215409 | 2.355272689 | 0.11900106 | 0.084198053 | 0.387520881 | 0.279038541 | 10.49546521 | 47.42390264 |
| 0.336394661 | 1.813283838 | 0.101257708 | 0.113325876 | 0.265096681 | 0.167745326 | 12.46695126 | 44.80953156 |
| 0.472906887 | 2.441621905 | 0.082684371 | 0.061260971 | 0.330176898 | 0.207511447 | 12.28818829 | 55.95548346 |
| 0.262573218 | 1.474449606 | 0 | 0 | 0.141385579 | 0.090890729 | 16.79458695 | 49.30317108 |
| 0.256726227 | 2.105155063 | 0 | 0.082152393 | 0.164304785 | 0.236188129 | 11.40891353 | 58.80057507 |
| 0.253421186 | 1.844906234 | 0 | 0 | 0.192600101 | 0.364926508 | 11.83983781 | 54.2524075 |
| 0.263504611 | 2.118171683 | 0 | 0.091213135 | 0.202695855 | 0.121617513 | 12.567143 | 54.93057667 |
| 0.315136729 | 2.094134391 | 0 | 0 | 0.376130934 | 0.386296635 | 19.97560232 | 49.78143743 |
| 0.300689194 | 2.376718267 | 0.160890659 | 0.033179561 | 0.284193309 | 0.392111947 | 11.69561411 | 47.60264514 |
| 0.294342262 | 2.50547287 | 0.195250303 | 0.134217121 | 0.343059844 | 0.356995917 | 12.38202463 | 42.78957709 |
| 0.261764186 | 2.39388025 | 0.136547451 | 0.080122443 | 0.383369756 | 0.217167116 | 11.40139009 | 44.35337076 |
| 0.230628456 | 2.282111614 | 0.0801373 | 0.072800401 | 0.29551791 | 0.173073168 | 11.6405004 | 52.93984378 |
| 0.419467535 | 2.770527667 | 0.057905686 | 0.075003156 | 0.274532789 | 0.169701953 | 11.46595524 | 50.52304504 |
| 0.305935142 | 2.926779523 | 0 | 0.112176219 | 0.193758923 | 0.101978381 | 17.38731389 | 51.90699572 |
| 0.202839757 | 1.825557809 | 0 | 0.182555781 | 0.14198783 | 0.070993915 | 19.7464503 | 50.18255578 |
| 0.252882865 | 1.58810439 | 0 | 0.101153146 | 0.121383775 | 0.050576573 | 20.97916245 | 49.36273518 |
| 0.182759671 | 2.132196162 | 0.071073205 | 0.111686466 | 0.416285917 | 0.233526246 | 17.37232206 | 48.85775206 |
| 0.2236909 | 3.324860193 | 0.101677682 | 0.091509914 | 0.284697509 | 0.264361973 | 16.06507372 | 48.44941535 |
| 0.392879883 | 3.01424319 | 0.325191852 | 0.216391815 | 0.345530878 | 0.725789276 | 18.28819422 | 42.30258033 |
| 0.311552775 | 2.648576975 | 0.140625587 | 0.098999838 | 0.29001395 | 0.362044028 | 13.34773341 | 42.14148591 |
| 0.256019455 | 2.584149018 | 0.096342297 | 0.177487759 | 0.341594797 | 0.210964403 | 22.49479462 | 44.7793728 |
| 0.295551329 | 2.259684657 | 0.081799093 | 0.156216663 | 0.325382189 | 0.172916281 | 21.00835746 | 46.68661838 |
| 0.349643932 | 2.080833027 | 0.0376531 | 0.025786409 | 0.258480326 | 0.166232538 | 21.27752098 | 44.33290363 |
| 0.242008672 | 2.541091056 | 0 | 0.080669557 | 0.231924977 | 0.121004336 | 18.93717858 | 50.70081678 |
| 0.379098361 | 3.329918033 | 0 | 0.12295082 | 0.379098361 | 0.297131148 | 13.54508197 | 54.21106557 |
| 0.468813698 | 3.189971464 | 0 | 0.101916021 | 0.591112923 | 0.315939666 | 16.8263351 | 50.80513657 |
| 0.28830313 | 2.985996705 | 0 | 0.267710049 | 0.27800659 | 0.226523888 | 13.0354201 | 54.53047776 |
| 0.207404335 | 3.43254174 | 0.103702167 | 0.362957586 | 0.95405994 | 1.099242974 | 17.33900239 | 47.16374572 |
| | | | | | | | |
| 0.260103643 | 3.540977682 | 0.074876746 | 0.123564306 | 0.246676357 | 0.347643522 | 12.87357864 | 42.4428289 |
| 0.317033946 | 3.172963988 | 0.083294681 | 0.078316678 | 0.45888255 | 0.352137881 | 16.55541186 | 45.16426544 |
| 0.343700608 | 1.800559375 | 0.074596095 | 0.163589402 | 0.196225325 | 0.124812966 | 21.14722897 | 43.26180308 |
| 0.379373931 | 3.404677743 | 0.099488674 | 0.108689145 | 0.52478976 | 0.33580411 | 14.59603377 | 46.74917839 |
| 0.401771917 | 3.883795199 | 0 | 0 | 0.53569589 | 0.288451633 | 12.81549397 | 52.68363037 |
| 0.277492292 | 2.137718397 | 0 | 0 | 0.184994861 | 0.143884892 | 14.16238438 | 52.56937307 |
| 0.677223384 | 2.718852704 | 0 | 0 | 0.288815855 | 0.776815058 | 14.96862862 | 48.899512 |
| 0.352538276 | 2.326752619 | 0 | 0.221595488 | 0.251813054 | 0.161160355 | 15.11885576 | 51.37993554 |
| 0.26793075 | 3.122423743 | 0.154575433 | 0 | 0.659521847 | 0.463726298 | 12.93281121 | 47.13520198 |
| 0.250660787 | 3.510561315 | 0.286100447 | 0.165523241 | 0.603610706 | 0.634544531 | 10.61154285 | 45.24001619 |
| 0.332888409 | 2.865564074 | 0.204473902 | 0.126974667 | 0.303693492 | 0.415357416 | 11.86789313 | 39.25518391 |
| 0.367044968 | 1.985613248 | 0.133218797 | 0.084788867 | 0.398035619 | 0.203745987 | 11.91309012 | 44.86443477 |
| 0.318098108 | 1.482447473 | 0.095891318 | 0.102582243 | 0.231128023 | 0.086256505 | 12.74805463 | 48.52788002 |
| 0.347669667 | 2.714555699 | 0.112491943 | 0.04719617 | 0.327629177 | 0.166205141 | 11.21281942 | 48.81941184 |
| 0.276526014 | 1.730848013 | 0 | 0.215075789 | 0.256042605 | 0.153625563 | 12.77140516 | 52.22244982 |
| 0.323725982 | 2.882205514 | 0 | 0 | 0.386382623 | 0.386382623 | 12.5 | 50.44903926 |
| 0.362468931 | 4.173570837 | 0 | 0 | 0.455675228 | 0.362468931 | 10.96727423 | 56.81441591 |
| 0.214132762 | 2.182114816 | 0 | 0.101967982 | 0.336494341 | 0.244723157 | 13.89823595 | 48.39400428 |
| 0.261431452 | 3.138720645 | 0.238458148 | 0.06526805 | 0.573595358 | 0.753520626 | 14.17496911 | 42.66183496 |
| 0.345783391 | 2.701096816 | 0.130448216 | 0.098073838 | 0.308833346 | 0.420871311 | 12.94377928 | 43.3967817 |
| 0.293795883 | 2.975370825 | 0.075844513 | 0.076770098 | 0.452050031 | 0.248896144 | 11.89662695 | 45.62486834 |
| 0.336431751 | 1.813483766 | 0.101268873 | 0.113338371 | 0.26512591 | 0.167763821 | 12.46832584 | 44.81447214 |
| 0.368204529 | 1.999473361 | 0.122835861 | 0.072506656 | 0.287630136 | 0.134600576 | 12.26923369 | 43.44276921 |

| total_n3 | total_PUFA | total_MUFA | total_SFA | n6:n3 ratio | M:S ratio | P:S ratio |
|---|---|---|---|---|---|---|
| 6.387456593 | 55.30884984 | 14.40597706 | 30.2851731 | 7.658978583 | 0.475677554 | 1.826268242 |
| 10.11178341 | 62.71151677 | 12.64485694 | 24.64362629 | 5.201825558 | 0.513108614 | 2.544735747 |
| 14.14895855 | 62.78140122 | 12.24489796 | 24.97370082 | 3.437174721 | 0.49031171 | 2.51390059 |
| 11.74692516 | 53.37711069 | 16.02042944 | 30.60245987 | 3.543921917 | 0.523501362 | 1.744209809 |
| 11.39993736 | 55.72606744 | 14.65706232 | 29.61687024 | 3.888278388 | 0.494888967 | 1.881565033 |
| | | | | | | |
| 6.050326379 | 48.18710948 | 16.63578287 | 35.17710765 | 6.96438183 | 0.472915028 | 1.369842852 |
| 12.48474442 | 51.1186539 | 14.12494437 | 34.75640173 | 3.094489417 | 0.406398352 | 1.470769451 |
| 13.81804415 | 56.21981787 | 12.96139232 | 30.81878981 | 3.068579987 | 0.420567855 | 1.824205889 |
| 13.10114794 | 56.20452624 | 13.75264386 | 30.0428299 | 3.290045918 | 0.457767924 | 1.870813316 |
| 7.241650737 | 57.28100753 | 15.00056224 | 27.71843023 | 6.909937888 | 0.541176471 | 2.06653144 |
| 8.702368692 | 60.67971164 | 13.62512873 | 25.69515963 | 5.972781065 | 0.530260521 | 2.361523046 |
| 8.651133633 | 57.74736182 | 14.85738167 | 27.3952565 | 5.675120773 | 0.542334096 | 2.107932876 |
| 10.03744539 | 61.19201165 | 12.8666528 | 25.94133555 | 5.096373057 | 0.495990377 | 2.358861267 |
| 6.864192324 | 56.07338676 | 16.42766765 | 27.49894559 | 7.168970814 | 0.597392638 | 2.039110429 |
| 10.21222427 | 54.18395618 | 13.68020311 | 32.13584072 | 4.305793796 | 0.425699244 | 1.686091136 |
| 8.264922975 | 48.86526838 | 17.73114842 | 33.4035832 | 4.912368274 | 0.530815761 | 1.462875048 |
| 9.476759562 | 52.73997627 | 13.04297548 | 34.21704825 | 4.56519092 | 0.381183537 | 1.541336233 |
| 9.309122831 | 61.99918604 | 3.750288459 | 34.2505255 | 5.660045974 | 0.109495793 | 1.810167439 |
| 12.71344617 | 55.01221101 | 14.4031626 | 30.58462639 | 3.327088836 | 0.470928185 | 1.798688345 |
| 6.23872679 | 58.4403183 | 12.89124668 | 28.66843501 | 8.367346939 | 0.449666913 | 2.038490007 |
| 7.368747416 | 64.68582059 | 15.48160397 | 19.83257544 | 7.778401122 | 0.780614904 | 3.261594581 |
| | | | | | | |
| 5.319796954 | 56.59898477 | 17.59390863 | 25.8071066 | 9.639312977 | 0.681746656 | 2.193154996 |
| 6.559901447 | 57.93039729 | 15.18324607 | 26.88635664 | 7.830985915 | 0.564719359 | 2.154639175 |
| | | | | | | |
| 6.801398796 | 51.18261105 | 15.25050079 | 33.56688816 | 6.525306571 | 0.454331683 | 1.524794637 |
| 8.366218865 | 55.7901215 | 12.57074677 | 31.63913173 | 5.668498924 | 0.397316427 | 1.763326566 |
| 6.688495285 | 51.49802684 | 15.41813763 | 33.08383552 | 6.69949363 | 0.466032351 | 1.556591793 |
| 8.781782548 | 64.737266 | 14.78827271 | 20.47446128 | 6.371768277 | 0.722278965 | 3.161854425 |
| 6.614825288 | 55.91799636 | 18.90527166 | 25.17673197 | 7.453435115 | 0.750902527 | 2.221018853 |
| 3.696857671 | 62.49743274 | 13.27788047 | 24.22468679 | 15.90555556 | 0.548113607 | 2.57990674 |
| 7.399898632 | 61.65230613 | 13.45159655 | 24.89609731 | 7.331506849 | 0.540309446 | 2.476384365 |
| 7.530151008 | 62.46072768 | 14.04682274 | 23.49244958 | 7.294751009 | 0.597929249 | 2.65875755 |
| 5.123513266 | 54.9049507 | 22.14089661 | 22.95415269 | 9.716269841 | 0.964570416 | 2.39193977 |
| 8.295816289 | 55.89846143 | 13.83372029 | 30.26781828 | 5.738150832 | 0.457043853 | 1.846795197 |
| 6.680128518 | 49.46970561 | 15.04268299 | 35.4876114 | 6.405502076 | 0.423885474 | 1.393999305 |
| 7.30156467 | 51.65493543 | 14.14918825 | 34.19587632 | 6.074502214 | 0.413768845 | 1.510560365 |
| 7.339514369 | 60.27935814 | 13.20742297 | 26.51321888 | 7.212990003 | 0.498144832 | 2.273558651 |
| 6.129473376 | 56.65251841 | 13.33124592 | 30.01623566 | 8.242640426 | 0.444134503 | 1.887395843 |
| 3.457067102 | 55.36406282 | 18.84560473 | 25.79033245 | 15.01474926 | 0.730723606 | 2.1466983 |
| 5.14198783 | 55.32454361 | 21.23732252 | 23.43813387 | 9.759368836 | 0.906101255 | 2.360450022 |
| 4.825005058 | 54.18774024 | 22.25369209 | 23.55856767 | 10.23060797 | 0.944611421 | 2.300128811 |
| 4.751751447 | 53.6095035 | 19.43344502 | 26.95705148 | 10.28205128 | 0.720903955 | 1.988700565 |
| 5.693950178 | 54.14336553 | 18.08845958 | 27.76817489 | 8.508928571 | 0.65140974 | 1.949835225 |
| 5.553237835 | 47.85581817 | 20.23459738 | 31.90958445 | 7.617642462 | 0.634122874 | 1.499731789 |
| 5.36986204 | 47.51134795 | 16.24188724 | 36.24676481 | 7.847778136 | 0.448092053 | 1.310774857 |
| 3.587021374 | 48.36639418 | 24.14805889 | 27.48554693 | 12.48372065 | 0.878572981 | 1.75970281 |
| 4.379482713 | 51.06610109 | 22.82152471 | 26.1123742 | 10.6603043 | 0.873973563 | 1.95562842 |
| 5.133785656 | 49.46668928 | 23.01472415 | 27.51858656 | 8.63551901 | 0.836333803 | 1.797573766 |
| 3.509125744 | 54.20994252 | 20.51023495 | 25.27982253 | 14.44827586 | 0.811328281 | 2.144395692 |
| 3.288934426 | 57.5 | 15.49180328 | 27.00819672 | 16.48286604 | 0.573596358 | 2.128983308 |
| 4.239706482 | 55.04484305 | 18.50794945 | 26.4472075 | 11.98317308 | 0.699807322 | 2.081310212 |
| 5.40568369 | 59.93616145 | 14.45634267 | 25.60749588 | 10.08761905 | 0.564535585 | 2.340570969 |
| 7.539147568 | 54.70289329 | 19.6411905 | 25.65591621 | 6.255845942 | 0.765561843 | 2.132174616 |
| | | | | | | |
| 5.53613575 | 47.97896465 | 15.36953083 | 36.65150452 | 7.666507979 | 0.419342426 | 1.30905853 |
| 3.894240663 | 49.0585061 | 18.50933949 | 32.43215441 | 11.59770783 | 0.570709526 | 1.512650238 |
| 4.087791179 | 47.34959426 | 24.06859377 | 28.58181196 | 10.58317345 | 0.842094749 | 1.656633747 |
| 5.472900445 | 52.22207883 | 16.67616412 | 31.10175704 | 8.541938386 | 0.536180773 | 1.67907166 |
| 5.995673226 | 58.6793036 | 14.14443185 | 27.17626455 | 8.786941581 | 0.520470053 | 2.159211524 |
| 5.29290853 | 57.8622816 | 16.11510791 | 26.02261048 | 9.932038835 | 0.619273302 | 2.223538705 |
| 7.190518873 | 56.09003087 | 15.59605617 | 28.31391296 | 6.800554017 | 0.550826592 | 1.98100598 |
| 7.433521354 | 58.81345689 | 16.71031426 | 24.47622885 | 6.911924119 | 0.682716049 | 2.402880658 |
| 6.131492168 | 53.26669415 | 15.5708986 | 31.16240725 | 7.687394958 | 0.499669312 | 1.709325397 |
| 4.548764456 | 49.78878064 | 13.17571576 | 37.0355036 | 9.945561399 | 0.355759055 | 1.344352737 |
| 6.193323501 | 45.44850741 | 14.32196812 | 40.22952447 | 6.338306712 | 0.356006398 | 1.129730167 |
| 6.802913035 | 51.6673478 | 14.33834805 | 33.99430414 | 6.594885829 | 0.421786779 | 1.519882495 |
| 6.548283967 | 55.07616399 | 15.1019645 | 29.82187151 | 7.410778192 | 0.506405659 | 1.846837948 |
| 5.759129037 | 54.57854088 | 12.98125986 | 32.44019926 | 8.476874112 | 0.400159683 | 1.682435439 |
| 4.823842687 | 57.0462925 | 15.13723884 | 27.81646866 | 10.82590234 | 0.544182622 | 2.050810015 |
| 4.939431913 | 55.38847118 | 14.86006683 | 29.75146199 | 10.21353066 | 0.499473499 | 1.861705862 |
| 4.587821044 | 61.40223695 | 12.71748136 | 25.88028169 | 12.38374718 | 0.491396559 | 2.37254902 |
| 6.903232385 | 55.29723667 | 16.21290915 | 28.48985419 | 7.010339734 | 0.569076593 | 1.940944882 |
| 6.559790507 | 49.22162547 | 16.88703107 | 33.89134346 | 6.50353619 | 0.498269745 | 1.452336215 |
| 5.615357342 | 49.01213904 | 15.65791368 | 35.32994728 | 7.728231536 | 0.443190972 | 1.387268955 |
| 4.515910957 | 50.1407793 | 14.02257542 | 35.83664529 | 10.10313728 | 0.39129152 | 1.399148243 |
| 6.689232741 | 51.50370488 | 15.41983759 | 33.07645753 | 6.69949363 | 0.466187698 | 1.557110668 |
| 7.820007485 | 51.26277669 | 15.0624656 | 33.6747577 | 5.555336013 | 0.447292472 | 1.522290885 |
