# Supplementary material for: Sex-Specific Effects of Diets High in Unsaturated Fatty Acids on Spatial Learning and Memory in Guinea Pigs
Source: PLoS One. 2015 Oct 15;10(10):e0140485. doi: 10.1371/journal.pone.0140485 (PMC4607430; doi:10.1371/journal.pone.0140485)
Supplement: S1 Table — (PDF) [file pone.0140485.s002.pdf]

S1 Table. Model statistics for behaviors of the spatial cognition test and saliva cortisol concentrations.

Response variable	Predictor	Statistics		
		df	F-statistic	p-value
Latency - Learning phase	Group	3,71	0.382	0.766
	Sex	1,71	0.006	0.940
	Day	2,142	0.775	0.463
	Group:Sex	3,71	0.436	0.728
	Group:Day	6,142	1.739	0.116
	Sex:Day	2,142	6.577	0.002
	Group:Sex:Day	6,142	2.121	0.050
Errors - Learning phase	Group	3,71	1.402	0.249
	Sex	1,71	3.648	0.060
	Day	2,142	0.070	0.932
	Group:Sex	3,71	1.914	0.135
	Group:Day	6,142	1.895	0.086
	Sex:Day	2,142	4.866	0.009
	Group:Sex:Day	6,142	2.374	0.032
Movement - Learning phase	Group	3,71	0.784	0.507
	Sex	1,71	1.069	0.305
	Day	2,142	1.789	0.171
	Group:Sex	3,71	0.678	0.569
	Group:Day	6,142	1.916	0.082
	Sex:Day	2,142	4.979	0.008
	Group:Sex:Day	6,142	2.013	0.068
Latency - Retention test	Group	3,71	1.507	0.220
	Sex	1,71	10.503	0.002
	Day	1,71	2.042	0.157
	Group:Sex	3,71	1.707	0.173
	Group:Day	3,71	1.027	0.386
	Sex:Day	1,71	9.457	0.003
	Group:Sex:Day	3,71	3.125	0.031
Errors - Retention test	Group	3,75	2.036	0.116
	Sex	1,74	0.166	0.685
	Day	1,78	7.744	0.007
	Group:Sex	3,71	0.368	0.776
	Group:Day	3,74	0.313	0.816
	Sex:Day	1,77	0.534	0.467
	Group:Sex:Day	3,71	1.081	0.363
Movement - Retention test	Group	3,71	1.209	0.313
	Sex	1,71	11.189	0.001
	Day	1,71	1.499	0.225
	Group:Sex	3,71	1.930	0.133
	Group:Day	3,71	0.641	0.591

	Sex:Day	1,71	11.004	0.001
	Group:Sex:Day	3,71	3.395	0.023
Saliva cortisol concentrations	Group	3,74	0.355	0.786
	Sex	1,74	12.95	<0.001
	Day	3,542	1.548	0.201
	Stage	1,542	0.382	0.537
	Group:Sex	3,71	0.583	0.628
	Group:Day	9,524	0.751	0.662
	Group:Stage	3,542	3.033	0.029
	Sex:Day	3,539	1.819	0.143
	Sex:Stage	1,542	29.288	<0.001
	Day:Stage	3,542	2.481	0.06
	Group:Sex:Day	9,515	0.938	0.492
	Group:Sex:Stage	3,533	1.042	0.373
	Group:Day:Stage	9,506	0.59	0.806
	Sex:Day:Stage	3,536	1.145	0.331

Bright model statistics indicate predictors that were removed during model simplification based on the AIC (values for each predictor at the last appearance in the model).
