# Supplementary material for: Sex-Specific Effects of Diets High in Unsaturated Fatty Acids on Spatial Learning and Memory in Guinea Pigs
Source: PLoS One. 2015 Oct 15;10(10):e0140485. doi: 10.1371/journal.pone.0140485 (PMC4607430; doi:10.1371/journal.pone.0140485)
Supplement: S2 Table — (PDF) [file pone.0140485.s003.pdf]

S2 Table. Plasma fatty acids (% on total fatty acids) for single male and female groups.

Fatty Acid	Group							
	Chia (n-3)		Walnut (n-6)		Peanut (n-9)		Control	
	males	females	males	females	males	females	males	females
**SFAs**								
13:0	0.15 ± 0.07	0.20 ± 0.11	0.15 ± 0.07	0.21 ± 0.09	0.08 ± 0.04	0.21 ± 0.08	0.25 ± 0.09	0.29 ± 0.08
14:0	0.51 ± 0.05	0.52 ± 0.08	0.55 ± 0.13	0.49 ± 0.09	0.47 ± 0.05	0.48 ± 0.06	0.57 ± 0.12	0.63 ±0.17
15:0	1.29 ± 0.33	1.31 ± 0.38	1.35 ± 0.41	1.24 ± 0.33	1.00 ± 0.25	1.17 ± 0.33	1.47 ± 0.41	1.54 ± 0.41
16:0	14.66 ± 0.55	14.80 ± 0.42	12.47 ± 1.73	12.81 ± 0.56	13.27 ± 0.66	13.79 ± 0.46	13.88 ± 1.30	14.88 ± 0.48
17:0	1.28 ± 0.20	1.21 ± 0.19	1.15 ± 0.19	1.26 ± 0.22	1.03 ± 0.21	1.58 ± 0.54	1.47 ± 0.24	1.36 ± 0.26
18:0	12.04 ± 0.41	11.63 ± 0.51	11.62 ± 0.73	11.52 ± 0.65	11.56 ± 0.33	11.20 ± 0.51	13.11 ± 1.17	12.28 ± 0.49
20:0	0.09 ± 0.03	0.13 ± 0.03	0.15 ± 0.02	0.11 ± 0.02	0.22 ± 0.04	0.17 ± 0.04	0.22 ± 0.06	0.13 ± 0.03
24:0	0.09 ± 0.03	0.08 ± 0.02	0.04 ± 0.02	0.06 ± 0.01	0.13 ± 0.05	0.16 ± 0.06	0.07 ± 0.03	0.08 ± 0.02
**MUFAs**								
14:1	0.23 ± 0.03	0.21 ± 0.04	0.21 ± 0.04	0.19 ± 0.03	0.16 ± 0.02	0.17 ± 0.02	0.21 ± 0.05	0.29 ± 0.03
16:1 n-7c	0.50 ± 0.08	0.63 ± 0.08	0.55 ± 0.11	0.52 ± 0.06	0.37 ± 0.05	0.55 ± 0.06	0.42 ± 0.08	0.67 ± 0.05
17:1	0.06 ± 0.03 ^{a}	0.10 ± 0.03 ^{a}	0.12 ± 0.02 ^{ab}	0.12 ± 0.02 ^{ab}	0.14 ± 0.02 ^{b}	0.16 ± 0.03 ^{b}	0.12 ± 0.04 ^{b}	0.18 ± 0.03 ^{b}
18:1 n-9t	0.04 ± 0.02 ^{a}	0.03 ± 0.02 ^{a}	0.12 ± 0.05 ^{b}	0.11 ± 0.05 ^{b}	0.15 ± 0.03 ^{b}	0.10 ± 0.04 ^{b}	0.08 ± 0.03 ^{ab}	0.07 ± 0.02 ^{ab}
18:1 n-7t	0.16 ± 0.07	0.22 ± 0.08	0.28 ± 0.11	0.20 ± 0.08	0.19 ± 0.08	0.21 ± 0.11	0.18 ± 0.07	0.28 ± 0.09
18:1 n-9c	12.43 ± 0.51 ^{a}	11.76 ± 1.26 ^{a}	12.39 ± 0.53 ^{a}	12.89 ± 0.92 ^{a}	18.41 ± 0.87 ^{b}	15.77 ± 0.89 ^{c}	12.53 ± 0.49 ^{a}	12.43 ± 0.33 ^{a}
18:1 n-7c	0.62 ± 0.20 ^{a}	0.47 ± 0.16 ^{a}	1.09 ± 0.05 ^{b}	0.99 ± 0.02 ^{b}	0.97 ± 0.04 ^{b}	0.95 ± 0.03 ^{b}	1.04 ± 0.06 ^{b}	1.03 ± 0.02 ^{b}
20:1 n-9	0.11 ± 0.03 ^{a}	0.12 ± 0.02 ^{a}	0.15 ± 0.05 ^{a}	0.11 ± 0.02 ^{a}	0.24 ± 0.03 ^{b}	0.22 ± 0.02 ^{b}	0.18 ± 0.06 ^{ab}	0.16 ± 0.04 ^{ab}
**PUFAs**								
18:2 n-6t	0.21 ± 0.04	0.19 ± 0.03	0.12 ± 0.05	0.14 ± 0.06	0.16 ± 0.02	0.16 ± 0.03	0.17 ± 0.03	0.18 ± 0.03
18:2 n-6c	41.88 ± 1.44 ^{a}	44.28 ± 1.50 ^{a}	47.56 ± 1.76 ^{b}	47.69 ± 1.61 ^{b}	43.87 ± 1.16 ^{a}	44.61 ± 1.50 ^{a}	44.58 ± 1.35 ^{a}	44.21 ± 1.51 ^{a}
18:3 n-6	0.05 ± 0.02 ^{a}	0.06 ± 0.03 ^{a}	0.13 ± 0.01 ^{b}	0.14 ± 0.01 ^{b}	0.09 ± 0.02 ^{ab}	0.12 ± 0.02 ^{ab}	0.08 ± 0.02 ^{ab}	0.10 ± 0.03 ^{ab}
20:2 n-6	0.30 ± 0.02	0.30 ± 0.03	0.28 ± 0.03	0.29 ± 0.02	0.28 ± 0.02	0.32 ± 0.03	0.36 ± 0.04	0.31 ± 0.02
20:3 n-6	0.06 ± 0.02	0.07 ± 0.03	0.07 ± 0.03	0.08 ± 0.03	0.06 ± 0.02	0.08 ± 0.04	0.07 ± 0.03	0.08 ±0.03
20:4 n-6	2.22 ± 0.12	2.48 ± 0.16	2.43 ± 0.19	2.20 ± 0.21	2.44 ± 0.18	3.04 ± 0.58	2.67 ± 0.23	2.62 ± 0.26
18:3 n-3	10.46 ± 1.03 ^{a}	8.36 ± 0.56 ^{a}	6.36 ± 0.39 ^{b}	6.03 ± 0.42 ^{b}	4.20 ± 0.23 ^{c}	3.94 ± 0.33 ^{c}	5.38 ± 0.34 ^{b}	5.07 ± 0.42 ^{b}
20:5 n-3	0.06 ± 0.02	0.08 ± 0.02	0.05 ± 0.02	0.06 ± 0.02	0.09 ± 0.03	0.05 ± 0.02	0.10 ± 0.03	0.07 ± 0.03
22:5 n-3	0.31 ± 0.04	0.43 ± 0.06	0.33 ± 0.04	0.27 ± 0.03	0.27 ± 0.03	0.43 ± 0.08	0.38 ± 0.05	037 ± 0.04
22:6 n-3	0.20 ± 0.04	0.28 ± 0.09	0.27 ± 0.05	0.25 ± 0.04	0.24 ± 0.06	0.36 ± 0.10	0.33 ± 0.07	0.32 ± 0.06
**Totals**								
total n-9	12.58 ± 0.52 ^{a}	11.91 ± 1.25 ^{a}	12.65 ± 0.53 ^{a}	13.12 ± 0.92 ^{a}	18.80 ± 0.89 ^{b}	16.10 ± 0.95 ^{c}	12.84 ± 0.48 ^{a}	12.65 ± 0.32 ^{a}
total n-6	44.71 ± 1.47 ^{a}	47.43 ± 1.40 ^{a}	50.59 ± 1.69 ^{b}	50.53 ± 1.55 ^{b}	46.90 ± 1.07 ^{a}	48.34 ± 1.49 ^{a}	47.94 ± 1.29 ^{a}	47.54 ± 1.61 ^{a}
total n-3	11.03 ± 1.00 ^{a}	9.15 ± 0.53 ^{a}	7.01 ± 0.40 ^{b}	6.61 ± 0.42 ^{b}	4.79 ± 0.24 ^{c}	4.77 ± 0.45 ^{c}	6.19 ± 0.27 ^{b}	5.83 ± 0.40 ^{b}
total PUFA	55.74 ± 1.59 ^{ac}	56.58 ± 1.30 ^{ac}	57.61 ± 1.82 ^{a}	57.14 ± 1.43 ^{a}	51.69 ± 1.01 ^{b}	53.11 ± 1.44 ^{bc}	54.13 ± 1.34 ^{bc}	53.36 ± 1.40 ^{bc}
total MUFA	14.16 ± 0.49 ^{a}	13.54 ± 0.19 ^{a}	14.90 ± 0.56 ^{a}	15.14 ± 0.95 ^{a}	20.63 ± 0.79 ^{b}	18.14 ± 1.01 ^{c}	14.81 ± 0.39 ^{a}	15.11 ± 0.40 ^{a}
total UFA	69.90 ± 1.21 ^{ab}	71.12 ± 1.08 ^{ab}	72.51 ± 1.88 ^{a}	72.28 ± 1.42 ^{a}	72.32 ± 1.22 ^{ab}	71.25 ± 1.30 ^{ab}	68.93 ± 1.57 ^{b}	68.47 ± 1.20 ^{b}
total SFA	30.10 ± 1.21 ^{ab}	29.88 ± 1.08 ^{ab}	27.50 ± 1.88 ^{a}	27.72 ± 1.42 ^{a}	27.68 ± 1.22 ^{ab}	28.75 ± 1.30 ^{ab}	31.07 ± 1.57 ^{b}	31.53 ± 1.20 ^{b}
**Ratios**								
n6:n3 ratio	4.46 ± 0.58 ^{a}	5.36 ± 0.37 ^{a}	7.36 ± 0.45 ^{b}	8.14 ± 0.93 ^{b}	10.10 ± 072 ^{c}	10.85 ± 1.08 ^{c}	7.89 ± 0.42 ^{b}	8.56 ± 0.79 ^{b}
M:S ratio	0.47 ± 0.01 ^{a}	0.46 ± 0.04 ^{a}	0.56 ± 0.05 ^{ac}	0.56 ± 0.05 ^{ac}	0.76 ± 0.05 ^{b}	0.64 ± 0.05 ^{bc}	0.49 ± 0.03 ^{a}	0.48 ± 0.02 ^{a}
P:S ratio	1.89 ± 0.13 ^{ab}	1.92 ± 0.10 ^{ab}	2.21 ± 0.24 ^{a}	2.12 ± 0.14 ^{a}	1.91 ± 0.10 ^{ab}	1.89 ± 0.12 ^{ab}	1.80 ± 0.13 ^{b}	1.73 ± 0.12 ^{b}
U:S ratio	2.37 ± 0.14 ^{ab}	2.39 ± 0.12 ^{ab}	2.77 ± 0.28 ^{a}	2.69 ± 0.18 ^{a}	2.67 ± 0.14 ^{ab}	2.53 ± 0.14 ^{ab}	2.29 ± 0.16 ^{b}	2.21 ± 0.13 ^{b}

Different superscripts indicate significant differences between groups ($p \leq 0.05$).
